# The Invasive Capacity of HPV Transformed Cells Requires the hDlg-Dependent Enhancement of SGEF/RhoG Activity

**DOI:** 10.1371/journal.ppat.1002543

**Published:** 2012-02-23

**Authors:** Vanitha Krishna Subbaiah, Paola Massimi, Siaw Shi Boon, Michael P. Myers, Lisa Sharek, Rafael Garcia-Mata, Lawrence Banks

**Affiliations:** 1 International Centre for Genetic Engineering and Biotechnology, Trieste, Italy; 2 Department of Cell and Developmental Biology, University of North Carolina, Chapel Hill, North Carolina, United States of America; University of Michigan, United States of America

## Abstract

A major target of the HPV E6 oncoprotein is the human Discs Large (hDlg) tumour suppressor, although how this interaction contributes to HPV-induced malignancy is still unclear. Using a proteomic approach we show that a strong interacting partner of hDlg is the RhoG-specific guanine nucleotide exchange factor SGEF. The interaction between hDlg1 and SGEF involves both PDZ and SH3 domain recognition, and directly contributes to the regulation of SGEF's cellular localization and activity. Consistent with this, hDlg is a strong enhancer of RhoG activity, which occurs in an SGEF-dependent manner. We also show that HPV-18 E6 can interact indirectly with SGEF in a manner that is dependent upon the presence of hDlg and PDZ binding capacity. In HPV transformed cells, E6 maintains a high level of RhoG activity, and this is dependent upon the presence of hDlg and SGEF, which are found in complex with E6. Furthermore, we show that E6, hDlg and SGEF each directly contributes to the invasive capacity of HPV-16 and HPV-18 transformed tumour cells. These studies demonstrate that hDlg has a distinct oncogenic function in the context of HPV induced malignancy, one of the outcomes of which is increased RhoG activity and increased invasive capacity.

## Author Summary

The E6 oncoproteins from cancer-causing Human Papillomaviruses (HPVs) all share the capacity to target cellular PDZ domain containing proteins. The first such target of E6 to be identified was the cell polarity regulator Discs Large (Dlg). However owing to the limited information on the molecular basis for hDlg function, there is currently no information on what the role of the HPV E6-Dlg interaction might mean for the development of cervical cancer. In this study we have identified the molecular basis by which Dlg can regulate cell migration and invasion. This involves an interaction with SGEF, which in turn results in enhanced levels of RhoG activity and hence increased cell invasive capacity. Most importantly, we show that HPV-18 and HPV-16 E6 maintain this activity of Dlg to enhance a cell's invasive potential. These studies have major implications for how E6-PDZ interactions might contribute to HPV induced malignancy. Furthermore they also provide compelling evidence to explain how Dlg can contribute to the regulation of cell invasion and migration, and indicate that certain cellular pools of Dlg have distinct oncogenic potential.

## Introduction

Human Papillomaviruses (HPVs) are the causative agents of cervical cancer, the second major cause of cancer-related death in women worldwide [1], [2]. This is brought about by the combined action of two viral oncoproteins, E6 and E7, which subvert cellular regulatory pathways controlling cell cycle and cell survival [3], [4], [5]. Whilst over 100 different HPV types have been identified, only a small subset have been defined as causative agents for the development of cervical cancer, and these types are collectively termed high risk. Amongst these, the most prevalent are HPV-16 and HPV-18, which account for approximately 80% of the cervical cancer burden [6], [7]. Critical cellular targets of the viral oncoproteins include p53 and the pRb family of tumour suppressors, which are subject to proteasome-mediated degradation [8], [9], [10]. However, other activities of both E6 and E7 are also required for their full transforming potential [11], [12]. A particularly intriguing class of substrates for the high risk HPV E6 oncoproteins are cellular proteins that contain PDZ (PSD95/Dlg/ZO-1) domains, since the capacity to interact with these cellular proteins is only found amongst those E6 proteins derived from the high risk virus types. To date, over 10 such PDZ domain-containing targets of E6 have been identified, and they have been implicated in processes ranging from control of cell polarity, cell-cell attachment and regulation of diverse cell signaling pathways [13].

The first PDZ domain-containing target of HPV-16 and HPV-18 E6 to be described was the human homologue of the Drosophila tumour suppressor protein Discs Large (hDlg) [14], [15], which was shown to be a target for E6-mediated degradation [16]. hDlg is a member of the membrane-associated guanylate kinase (MAGUK) family of scaffolding proteins, being found at adherens junctions in epithelial cells and synaptic junctions in neurons, where it is required for the correct formation of both types of cell junctions [17]–[19]. At these sites it is believed to act as a scaffold that functions by clustering ion channels [20], [21], mediating trafficking of cell surface receptors [22] and organizing signal transduction pathways through interaction with several proteins such as the actin-associated proteins of the protein 4.1/ERM (Ezrin, Radixin, Moesin) superfamily [23]–[24], [25]–[26], calcium dependent Calmodulin [27], vinexin, β-catenin [28], and Net1, a RhoA specific GEF [29].

Numerous studies in *Drosophila* have also shown that mutations in DLG result in defects in cell polarity and tissue organization, ultimately resulting in tissue overgrowth and a malignant-like phenotype [17]–[19], [30], indicating a possible tumour suppressor function in mammalian cells [31]. Although hDlg has been implicated directly in the regulation of cell polarity and cell-cell attachment in mammalian cells [32], its role as a tumour suppressor is still unclear. Compelling indirect evidence comes from studies showing that it is also a target for other viral oncoproteins, including the Adenovirus 9 E4ORF1 and the HTLV-1 Tax oncoproteins [33], [34]. In addition, analysis of hDlg expression in different human tumours, including cervical, colon and breast cancers, indicates a frequent loss of protein expression during the later stages of malignancy, which is also consistent with a tumour suppressor function [35], [36]. However, there are also reports that certain isoforms of hDlg, when subverted by Adenoviral oncoproteins, may actually possess oncogenic activity [33]. Furthermore, although hDlg expression is lost during the later stages of cervical cancer progression, in many high-grade lesions and cervical tumour-derived cell lines there are still very high levels of Dlg expression, albeit often mislocalised [35].

The molecular basis underlying hDlg function in tumourigenesis is still largely obscure and its role, if any, in the development of HPV-induced malignancy is still unclear. hDlg is subject to different post- translational modifications, including being a substrate for ERK5, CDK1, and CDK2, with the latter being implicated in its ability to contribute to the control of cell cycle [37], [38]. In addition, hDlg is also phosphorylated by the MAPKs, p38γ and JNK, which control hDlg subcellular distribution and susceptibility to E6 targeting [39], [40]. Thus, whilst hDlg is a substrate for E6 induced degradation, it is also clear that only certain cellular pools of the protein are degraded, and significant levels of hDlg can still be detected in HPV positive tumour cells [41], [40], [38].

As a means of better understanding the role of hDlg in HPV-induced malignancy, we have performed a proteomic analysis to identify hDlg-interacting partners. We now show that a specific RhoG guanine nucleotide exchange factor (GEF), SGEF, is a strong interacting partner of hDlg and a consequence of this interaction is enhanced RhoG activity. We also show that a discrete pool of Dlg is maintained in complex with E6 and SGEF in HPV-18 positive cervical tumour-derived cells, which in turn contributes to maintaining high levels of RhoG activity and thus directly contributes to the invasive potential of these cells.

## Results

### SGEF is a novel interacting partner of hDlg

**Figure 1:**
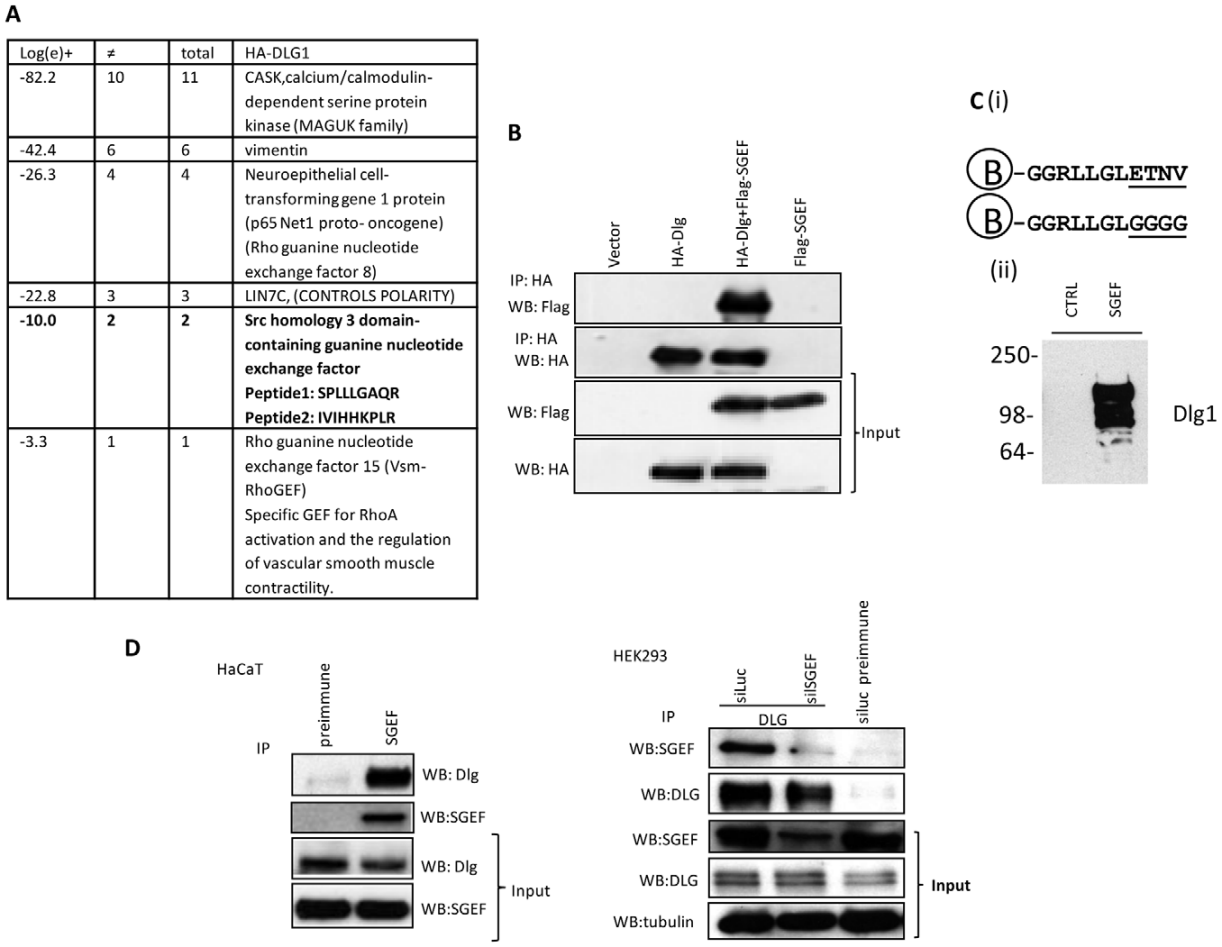
Identification of SGEF as an interacting partner of Dlg. Panel A. HEK293 cells were transfected with HA-tagged Dlg expression plasmid and after 24 hrs cells were immunoprecipitated using anti-HA antibody-conjugated agarose beads. The total protein complex was then subjected to mass spectroscopy analysis. The table shows a selection of the prominent Dlg interacting proteins, together with the peptide sequences showing SGEF as a novel interacting partner. Panel B. HEK293 cells were transfected with HA-tagged Dlg and Flag-tagged SGEF expression plasmids, as indicated, and after 24 hrs cell extracts were immunoprecipitated using anti HA-antibody conjugated agarose beads. Dlg-bound SGEF was then detected by western blotting using anti-Flag antibody. Panel C. Mouse liver lysate was incubated with streptavidin-Sepharose beads coupled with biotinylated peptides corresponding to the last 10 amino acids of SGEF or control (CTRL) peptides (i) in which the last 4 amino acids were replaced by glycine (underlined). (ii) After being washed, the samples were separated by SDS-PAGE and immunobloted with anti-Dlg1 antibodies. Panel D. HaCaT and HEK293 cell extracts were subjected to immunoprecipitation with pre-immune, anti-SGEF or anti-Dlg antibody as indicated, and the corresponding co-immunoprecipitating Dlg or SGEF detected by western blotting as indicated. As an additional control, HEK293 cells were also transfected with siRNA SGEF or siRNA Luc 72 hrs prior to harvesting, thereby confirming the identity of the SGEF protein detected with the anti SGEF antibody.

We performed a proteomic screen for Dlg interacting partners by transfecting HA-tagged Dlg into HEK293 cells (where endogenous hDlg levels are low) and subjecting the cell extracts to immunoprecipitation using anti-HA antibody-conjugated agarose beads. Total immunoprecipitates were then subjected to mass spectroscopic analysis and the resulting protein profiles were compared with those obtained from mock-transfected cells to exclude non-specific interactions. As can be seen from Figure 1A, a number of previously reported interacting partners of hDlg were identified in this analysis, including Net1, CASK and LIN7C, confirming these as *bona fide* interacting partners of hDlg [29], [42]. Amongst the novel potential interacting partners identified, we selected Src homology 3 domain-containing guanine nucleotide exchange factor (SGEF) as a particularly interesting candidate. This protein is a RhoG specific GEF, and has been reported to play important roles in regulating cell-cell contact and cell migration [43]. Furthermore, SGEF also contains a PDZ binding motif (PBM) at its C-terminus, suggesting one means by which it might interact with hDlg. To confirm that Dlg and SGEF can interact, HA-tagged Dlg was over-expressed either alone or in combination with Flag-tagged SGEF, and after 24 hrs the cells were harvested and cellular extracts were immunoprecipitated using anti-HA conjugated agarose beads. Co-immunoprecipitated SGEF was detected by western blot using anti-Flag antibody. The results in Figure 1B show that SGEF is specifically co-immunoprecipitated with Dlg, in agreement with the proteomic analyses. To determine whether the isolated SGEF PBM could recognize Dlg, a peptide pull-down assay was performed using either a biotinylated peptide corresponding to the carboxy terminal 10 amino acids of SGEF or a control (CTRL) peptide in which the last 4 amino acids, corresponding to the PBM consensus, were replaced with glycine residues (Figure 1C (i)). Immobilised peptides were then incubated with a mouse liver lysate and bound Dlg was detected by western blotting. The results in Figure 1C (ii) clearly show that the isolated SGEF PBM can specifically interact with Dlg. To further determine whether endogenously expressed hDlg and SGEF can exist in a complex we performed a series of co-immunoprecipitation experiments in HEK293 and HaCaT epithelial cells. In order to confirm the correct identity of the SGEF band that we were detecting, we also transfected an siRNA to SGEF in the HEK293 experiment. The results in Figure 1D show clear complex formation between hDlg and SGEF in both cell types.

### The interaction between Dlg and SGEF involves both PDZ and SH3 domains

**Figure 2:**
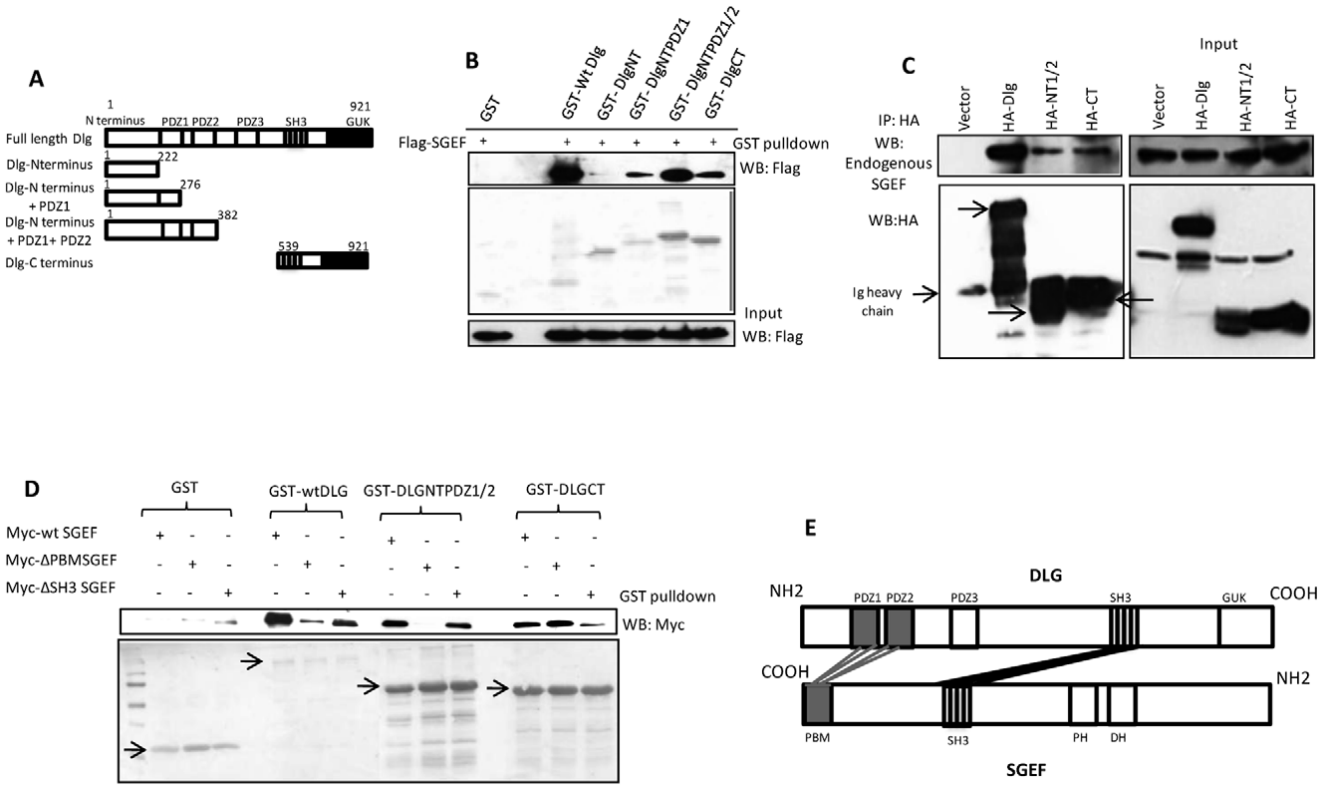
The interaction between Dlg and SGEF involves PBM-PDZ and SH3-SH3 domain recognition. Panel A. Diagrammatic representation of the Dlg deletion mutants used in this study. Panel B. HEK 293 cells were transfected with Flag-tagged SGEF expression plasmid and cell extracts made after 24 hrs. These were then incubated with a panel of GST.Dlg fusion proteins encompassing different domains of the Dlg protein and GST alone as a negative control. Bound SGEF was then detected by western blotting using anti-Flag antibody (upper panel). The middle panel shows the Ponceau stain of the membrane confirming similar levels of GST protein expression, whilst the bottom panel shows the input of SGEF used in each assay. Panel C. HEK293 cells were transfected with HA-tagged wild type Dlg and two mutants encompassing the N and C terminal halves of the protein. After 24 hrs the cells were extracted and Dlg precipitated using anti-HA antibody. The co-immunoprecipitated endogenously expressed SGEF was then detected by western blot using anti-SGEF antibody (upper left panel). Input proteins are shown in the two right panels and the lower left panel shows the immunoprecipitated Dlg (indicated by arrows). Panel D. HEK293 cells were transfected with myc-tagged wild type SGEF and mutants deleted in the PDZ binding motif and the SH3 domain. After 24 hrs cell extracts were made and incubated with purified GST fusion protein, consisting of GST alone, wild type Dlg and the N and C terminal halves of Dlg. Bound SGEF was detected by western blot using anti-myc antibody (upper panel). The lower panel shows the Ponceau stain of the membrane demonstrating the levels of GST protein expression with arrows indicating the relevant full-length GST fusion proteins. Panel E. Schematic summarizing the results of the interaction assays, demonstrating interaction between the Dlg PDZ1 and PDZ2 domains with the SGEF PBM plus association between the SGEF and Dlg SH3 domains.

To investigate further the mechanism of association between Dlg and SGEF, a series of purified GST.Dlg fusion proteins (Figure 2A) were used in pull-down assays of cell extracts from HEK293 cells that had been transfected with a plasmid expressing Flag-tagged SGEF. Bound SGEF was then detected by western blot with anti-Flag antibodies. The results in Figure 2B show a very strong interaction between wild type (wt) full-length Dlg and SGEF, whilst no interaction was obtained with the NT region (residues 1–222) of Dlg. In contrast, strong interaction was obtained with a construct encompassing PDZ domains 1 and 2 (residues 1–382), with a somewhat weaker interaction with a construct encompassing PDZ domain 1 (residues 1–276). Interestingly, a significant degree of association was also obtained with the carboxy terminal region of Dlg (residues 539–921). These results suggest that SGEF binds to sites within the PDZ domains of Dlg and also within the carboxy terminus, which contains an SH3 domain. As a further confirmation of these experiments, HEK293 cells were transfected with different HA-tagged constructs of Dlg, then the cell extracts were immunoprecipitated using anti-HA conjugated agarose beads and endogenously bound SGEF was detected in western blots using anti-SGEF antibody. The results shown in Figure 2C demonstrate strong co-immunoprecipitation between wt Dlg and SGEF with reduced, but still significant, levels of interaction between mutants of Dlg and SGEF that encompass just the N-terminal PDZ domains and the carboxy terminus of Dlg.

The above results indicate that SGEF can recognize two different sites on Dlg: the PDZ and SH3 domains. To investigate this further, pull-down assays were again performed using purified GST.Dlg fusion proteins and extracts from cells transfected with Myc-tagged wt SGEF and mutants of SGEF deleted in the PBM and SH3 domains. Bound SGEF was detected by western blot with anti-Myc antibody. The results in Figure 2D again show strong interaction between wt SGEF and wt Dlg, but SGEF mutated in the PBM or SH3 domains shows reduced but still significant levels of interaction. Interestingly, a Dlg mutant that lacks the SH3 domain, but retains two PDZ domains (DLGNT1/2), still binds strongly to an SGEF mutant that retains the PBM but lacks the SH3 domain, whilst no interaction is seen with the SGEF PBM mutant. In contrast, a Dlg mutant that only contains the SH3 domain binds strongly to an SGEF mutant that lacks the PBM, but not to an SGEF mutant that is deleted in the SH3 domain. Taken together these results demonstrate that the Dlg-SGEF interaction involves both PDZ and SH3 domain recognition, and this is summarized schematically in Figure 2E.

### Dlg regulates SGEF localization

**Figure 3:**
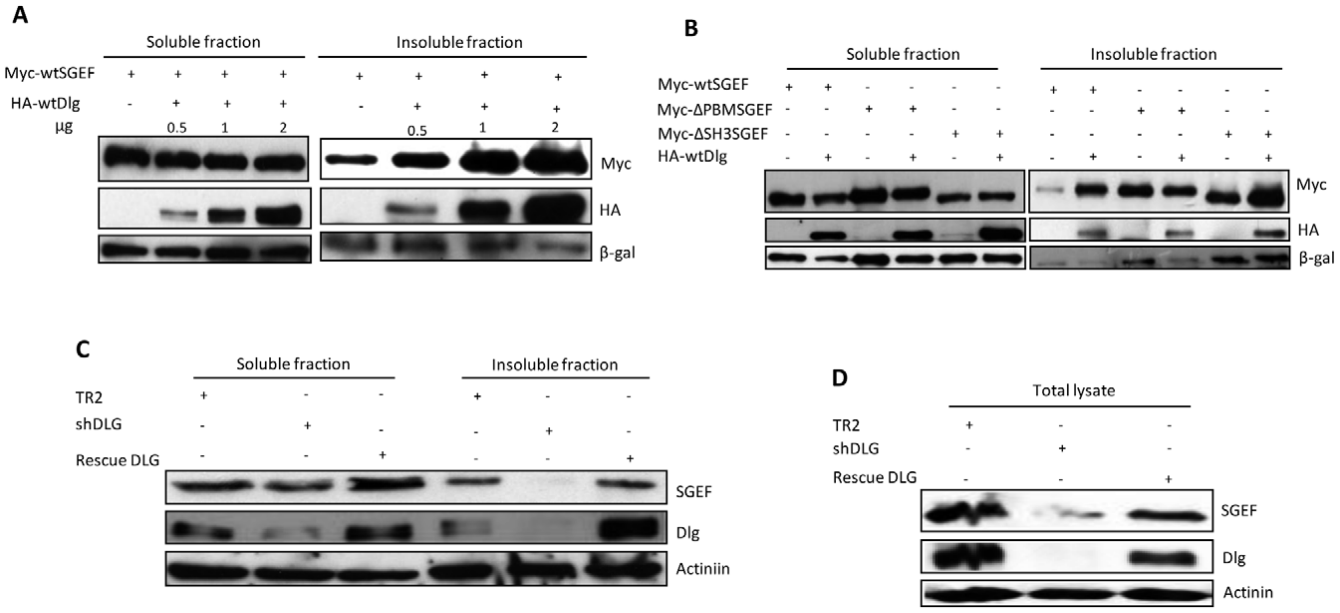
Dlg regulates the cellular localisation of SGEF. Panel A. HEK293 cells were transfected with myc-tagged SGEF expression plasmid together with increasing amounts of HA-tagged Dlg expression plasmid. After 24 hrs cells were extracted into soluble (left panel) and insoluble (right panel) fractions. Changes in the patterns of SGEF localisation were detected by western blot using anti-Myc antibody and Dlg was detected using anti-HA antibody. The levels of β-gal expression are also shown as a marker for transfection efficiency and loading control. Panel B. HEK293 cells were transfected with the Myc-tagged SGEF expression plasmids together with a HA-tagged Dlg expression plasmid. After 24 hrs the cells were extracted into soluble and insoluble fractions and the pattern of expression determined by western blot analysis. β-gal expression was used as a marker for transfection efficiency and a loading control. Panel C. HaCaT cells stably selected with control targeting plasmid (TR2), shDlg targeting plasmid, and cells targeted for shDlg but rescued with rat Dlg were analysed for the pattern of SGEF expression in the soluble and insoluble fractions by western blotting with anti-SGEF antibody. α-Actinin was used as a loading control. Note the complete loss of expression of SGEF from the insoluble fraction of the cell upon hDlg ablation. Panel D. Western blot analysis of SGEF expression levels in the total cell lysates prepared from stable cell lines used in Panel C.

Previous studies have shown that hDlg has many characteristics of a molecular scaffold, coordinating the assembly of multi-protein complexes within defined cellular compartments [44]. Having confirmed the interaction between DLG and SGEF we wanted to determine whether Dlg could modulate the pattern of SGEF expression. To do this, HEK 293 cells were transfected with Myc-tagged SGEF alone or in combination with increasing amounts of plasmid expressing HA-tagged Dlg. After 24 hrs, NP-40 soluble and insoluble cellular extracts were prepared, and the pattern of SGEF expression was analysed by western blot using anti-Myc antibody. The results in Figure 3A demonstrate that Dlg appears to have no major effect on the NP-40 soluble pool of SGEF, but increases the amount of SGEF in the NP-40 insoluble pool quite dramatically. Since we have shown that the association between the Dlg and SGEF involves both PDZ and SH3 domain recognition, we next investigated which of these interactions was responsible for this phenotype. To do this we repeated the assay, but included mutants of SGEF deleted in the PBM or SH3 domain. The results in Figure 3B again show that Dlg has no significant effect upon the levels of SGEF expression in the NP-40 soluble fraction of the cell, whilst wt SGEF increases dramatically in the insoluble portion. Interestingly, Dlg has minimal effect upon the solubility of the SGEF ΔPBM mutant, whereas the ΔSH3 mutant of SGEF behaves like the wt protein, and there is a significant increase in the NP-40 insoluble pool of this mutant in the presence of Dlg. Both the ΔPBM and ΔSH3 mutants display significant levels of expression within the NP-40 insoluble fraction in the absence of ectopic Dlg, and this degree of mislocalisation, which has been reported previously [45], may be related to their relatively increased levels of expression. However, taken together these results demonstrate that although the interaction between Dlg and SGEF involves the PDZ and SH3 domains, it is only the PBM-PDZ domain interaction that affects the pattern of SGEF expression in response to ectopically expressed Dlg. To further investigate the effects of hDlg upon SGEF, we analyzed SGEF levels in epithelial cells where hDlg expression was stably ablated, and compared this with control cells and cells in which targeting vector-resistant rat Dlg was re-expressed. The results in Figure 3C show that loss of hDlg results in a dramatic decrease in total SGEF levels, most particularly in the NP-40 insoluble fraction of the cell. Similar effects of Dlg on SGEF were observed in the total lysates (Figure 3D), and in both cases re-introduction of rat Dlg restored the levels of SGEF expression.

**Figure 4:**
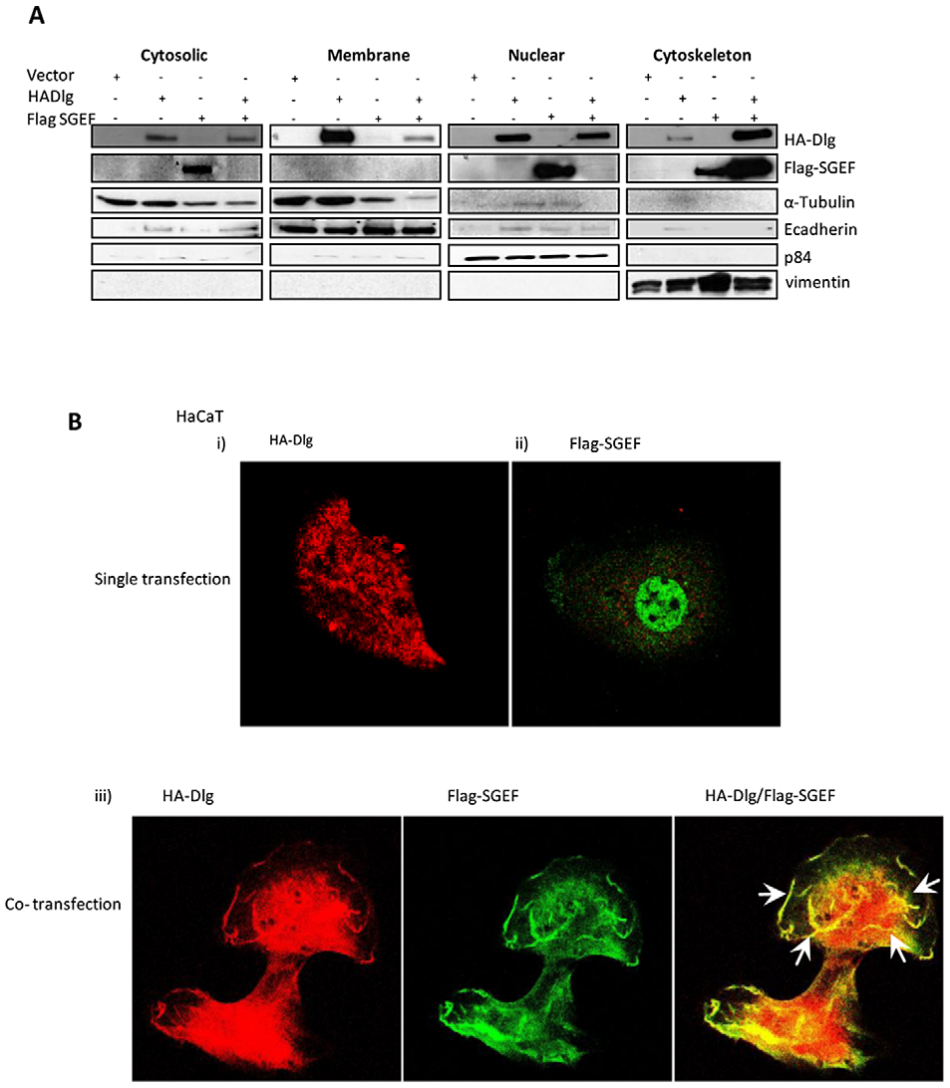
Dlg recruits SGEF to the cytoskeletal network. Panel A. HEK293 cells were transfected with HA-tagged Dlg and Flag-tagged SGEF expression plasmids as indicated and after 24 hrs cells were extracted and divided into cytosolic, membrane, nuclear and cytoskeletal fractions. The pattern of Dlg and SGEF expression was then determined by western blotting with anti-HA and anti-Flag antibodies respectively. Loading controls confirming the integrity of the differential extractions are α-tubulin, E-cadherin, p84 and vimentin for the cytosolic, membrane, nuclear and cytoskeletal fractions respectively. Panel B. HaCaT cells transfected with HA-Dlg, Flag-SGEF or co-transfected with the two cDNAs were fixed and processed for immunofluorescence with anti-Flag to detect SGEF and anti-HA to detect Dlg. i) Single HA-Dlg transfection. ii) Single Flag-SGEF transfection. iii) Co-transfection with Dlg in red and SGEF in green. The arrows indicate discrete areas of co-localisation within cytoplasmic and membrane sites.

To determine the particular cellular compartment to which SGEF was being recruited by Dlg, HEK293 cells were transfected with expression plasmids encoding HA-tagged Dlg and Flag-tagged SGEF. Cell fractionations were performed to divide the extracts into cytosolic, membrane, nuclear and cytoskeletal pools. Patterns of Dlg and SGEF expression were then ascertained by western blotting and the results are shown in Figure 4A. As can be seen, in the presence of ectopically expressed Dlg there is a clear redistribution of SGEF from cytosolic and nuclear fractions into the cytoskeletal fraction of the cell. We wanted to further confirm the co-localization of DLG and SGEF by immunofluorescence. HaCaT cells were transfected with HA-DLG and Flag-SGEF, either alone or in combination. After 24 hrs, the cells were fixed and stained for DLG and SGEF. The results in Figure 4B and Figure S1 show that over expressed SGEF has a diffuse pattern of expression, with significant amounts present within the nucleus. However when Dlg was co-transfected with SGEF, there was a marked decrease in the amount of SGEF in the nucleus, and a concomitant increase within the cytoplasm, with both Dlg and SGEF showing a high degree of co-localisation within the cytoplasmic cytoskeleton.

### The interaction between Dlg and SGEF enhances endogenous RhoG activity

**Figure 5:**
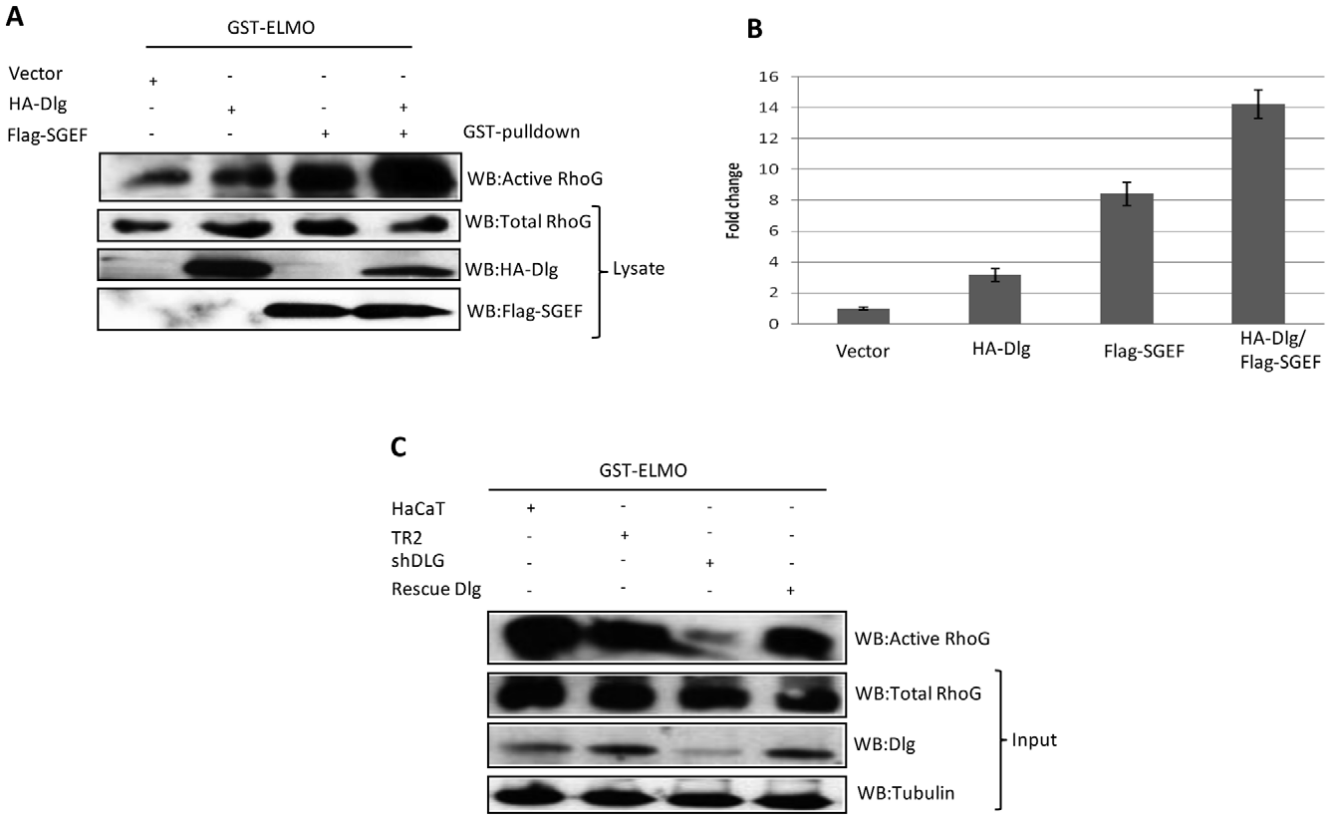
Dlg enhances RhoG activity in an SGEF dependent manner. Panel A. HEK293 cells were either transfected with vector or HA-tagged Dlg and Flag-tagged SGEF as indicated. After 24 hrs cell extracts were made which were then incubated with purified GST-ELMO to pull down active RhoG which was detected by western blot analysis. The three lower panels show total protein inputs for RhoG, Dlg and SGEF. Panel B. Graph showing the quantification from multiple GST-ELMO pull-downs, showing the fold change in the levels of RhoG activity under the different experimental conditions. Error bars represent ±SD of three independent experiments Panel C. Extracts from HaCaT cells stably ablated for hDlg expression (shDlg) either with or without Dlg rescue expression were used in a GST-ELMO pulldown assay to determine RhoG activity. Extracts from untreated HaCaT cells or HaCaT cells stably expressing control shRNA(TR2) were used as control.

SGEF has been shown to be a GEF specific for RhoG [45]. To test whether the interaction and the recruitment of SGEF to the cytoskeleton by Dlg has any biological consequences in terms of SGEF regulation of RhoG, we proceeded to monitor the levels of RhoG activity, as determined by its ability to interact with the downstream target, ELMO [46]. To do this we performed an affinity pulldown that specifically precipitates active GTP-bound RhoG, as previously described [45]. The results obtained in Figure 5A, together with the quantifications from multiple assays in Figure 5B, show no alteration in total RhoG levels under the different assay conditions, whilst there is a significant increase in the levels of ELMO-bound active RhoG in cells transfected with SGEF, and this is in agreement with previous studies [45]. However, most strikingly, co-transfection of Dlg and SGEF results in a dramatic increase in the amount of active RhoG, suggesting that Dlg can increase SGEF activity with respect to its downstream effector, RhoG. As an additional verification of these studies, we also monitored RhoG activity in a similar manner in cells in which hDlg expression had been stably ablated. The results of the pull-down assays are shown in Figure 5C and clearly show that, whilst there are no changes in total levels of RhoG expression, there is a greatly reduced level of active RhoG present within the hDlg-depleted cells in comparison with the different sets of control cells, and this increases in the rescued rat Dlg-expressing cells. In conclusion, these data demonstrate that hDlg positively regulates SGEF- induced RhoG activity in epithelial cells.

### HPV18 E6/E7 maintains high levels of RhoG activity in a Dlg/SGEF dependent manner

**Figure 6:**
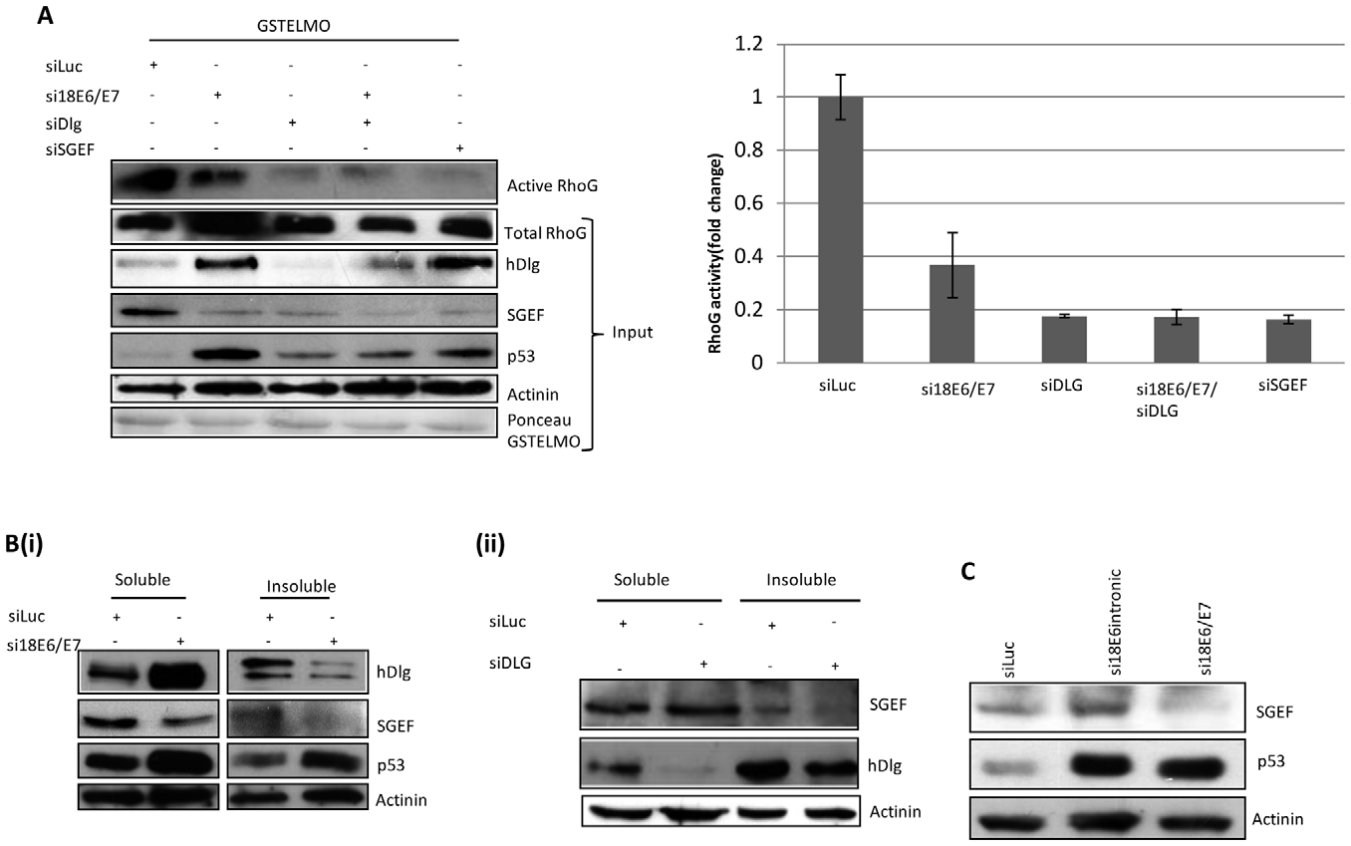
High levels of RhoG activity in HPV-18 transformed cells is hDlg and HPV -dependent. Panel A. HeLa cells were transfected with siRNAs against luciferase (Luc) control, E6/E7, hDlg or SGEF as indicated. After 72 hrs cell extracts were then incubated with purified GST.ELMO to determine the levels of active RhoG, which was detected by western blotting (upper panel). The lower panels show input levels of total RhoG, hDlg, SGEF, p53 and α-Actinin. Also shown is the Ponceau stain of the membrane showing constant levels of GST.ELMO. The graph shows the quantifications from multiple GST.ELMO pull-downs, and shows the fold change in the levels of RhoG activity under the different experimental conditions. Error bars represent +/− SD of four independent experiments. Note the modest decrease in active RhoG following the removal of E6/E7 and the dramatic decrease following removal of hDlg. Panel B. HeLa cells were transfected with control siRNA (Luc) siRNA to E6/E7 (i) or siRNA to hDlg-1 (ii) and were then analysed for the levels of hDlg, SGEF, and p53 expression after 72 hrs in the insoluble and soluble compartments of the cell. α-Actinin was used as a loading control. Note the marked increase in hDlg levels in the soluble fraction with concomitant decrease in the insoluble fraction following E6/E7 removal, which is also accompanied by a decrease in SGEF levels in this compartment and a similar loss of SGEF in the insoluble compartment is seen following siRNA ablation of hDlg-1 expression. Panel C. HeLa cells were transfected with control siRNA (Luc), siRNA to E6 (si18E6intronic) or siRNA to E6/E7 and were then analysed for levels of expression of SGEF and p53 in total cell lysates after 72 hrs. α-Actinin was used as a loading control.

Having shown that hDlg influences the activity of SGEF and RhoG, we wanted to determine what effects HPV-18 E6 might have upon this. To do this we first analysed the levels of RhoG activity in HPV-18 positive cervical tumour derived HeLa cells. GST-ELMO pull-downs were done using extracts from cells in which E6/E7 and hDlg1 expression had been ablated by siRNA transfection. The results and quantitations from multiple assays in Figure 6A show a number of interesting features. Firstly, RhoG activity levels are constitutively high in the control transfected cells. Ablation of E6/E7 expression results, as expected, in an increase in the total levels of hDlg expression and also, most surprisingly, results in a concomitant decrease in the levels of active RhoG. However, ablation of the residual hDlg alone, by siRNA transfection or in combination with siRNA E6/E7 also results in a dramatic decrease in the levels of active RhoG. These results suggest that high levels of RhoG activity in HeLa cells are dependent upon the presence of both E6/E7 and hDlg, even though certain pools of hDlg are also degraded by the HPV-18 E6 oncoprotein. This would therefore suggest that E6 is potentially recruiting or preserving a pool of hDlg that contributes to maintaining elevated SGEF activity. To investigate this possibility we analysed the levels of hDlg expression in the NP-40 soluble and insoluble pools of HeLa cells following ablation of E6/E7 expression. The results in Figure 6B(i) show that, as expected, there is a significant increase in the levels of hDlg expression in the NP-40 soluble fraction upon removal of E6//E7 expression. However, in the insoluble fraction there is a significant decrease in the levels of both hDlg and SGEF expression, suggesting that whilst certain pools of hDlg are targeted for degradation by E6, others are either actively maintained or unaffected. We also wanted to determine if the pattern of SGEF expression in other HPV positive cells was similarly dependent upon E6/E7 expression. To do this we analysed HPV-16 positive CaSki cells. The results in Figure S2 confirm that loss of E6/E7 expression also results in a loss of SGEF from the NP-40 insoluble pool in these cells.

To further verify that the existence of the NP-40 insoluble pool of SGEF was dependent upon the presence of hDlg, we performed an siRNA Dlg transfection in HeLa cells and the results in Figure 6B(ii) show that whilst loss of hDlg has a minimal effect upon the levels of SGEF in the NP-40 soluble fraction of the cell, there is a complete loss of SGEF from the NP-40 insoluble pool. Taken together these results imply that these NP-40 insoluble pools of hDlg and SGEF may possess properties that are favourable for maintenance of the malignant phenotype.

It is noteworthy from these experiments that loss of E6/E7 also appears to result in a general overall decrease in the levels of SGEF expression (Figures 6A and 6B(i)). Previous studies have shown that SGEF is responsive to regulation by E2F-1 [47], and we reasoned that loss of SGEF expression might in part be due to an indirect effect of E7 affecting the levels of E2F-1 activity via its association with pRb [8], [48]. To investigate this we repeated the assay but used two different siRNAs, one directed against just the HPV-18 E6, with the other against both E6 and E7. The results in Figure 6C show that loss of E6 alone has minimal effect on total SGEF levels, whilst loss of both E6 and E7 results in a decrease in SGEF expression, consistent with the notion that high levels of SGEF expression are being maintained in part by the E7 oncoprotein.

### HPV-18 E6 can exist in complex with hDlg1 and SGEF

**Figure 7:**
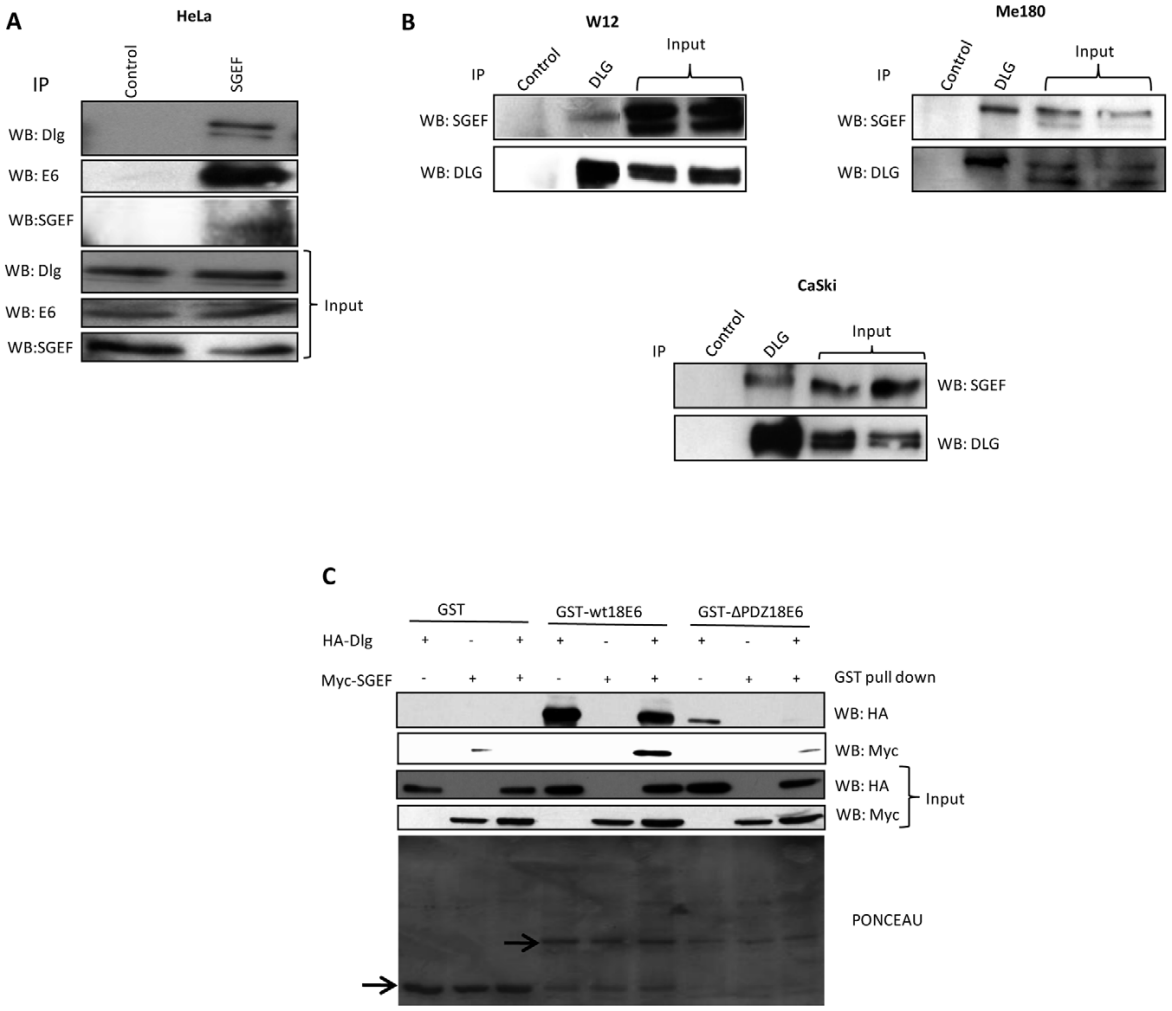
HPV-18E6, hDlg and SGEF exist as a complex. Panel A. HeLa cells were seeded in 10 cm^2^ dishes. After 24 hrs, cell extracts were prepared and immunoprecipitated using either the control antibody or the SGEF antibody. hDlg and HPV18E6 bound to the SGEF were detected using the anti-Dlg and anti-E6 antibodies respectively. The immunoprecipitated SGEF was detected using anti-SGEF antibody. The bottom 3 lanes show the input levels for hDlg, HPV18E6 and SGEF used in this assay. Panel B. HPV positive W12, Me180 and CaSki cells were seeded on 10 cm^2^ dishes and cellular extracts immunoprecipitated using either control antibody or anti-hDlg-1 antibody. The Dlg bound SGEF was detected by western blotting with the anti-SGEF antibody and the immunoprecipitated Dlg was detected using the anti-Dlg antibody. The right two lanes in each panel show the input levels (20%) of SGEF and Dlg used in the control and Dlg immunoprecipitates. Panel C. HEK 293 cells were transfected with HA-tagged Dlg and Myc-tagged SGEF expression plasmids either alone or in combination and the cell extracts made after 24 hrs. These were then incubated with GST-wt18E6 or GST-ΔPDZ18E6 and GST alone as a negative control. Bound Dlg was then detected by western blotting using anti-HA antibody (upper panel), bound SGEF was detected using anti-Myc antibody (second panel). The third and fourth panels shows the input of Dlg and SGEF used in this assay. The bottom panel shows the Ponceau stain of the membrane showing the levels of GST protein expression.

To determine whether the residual Dlg protein in HeLa cells was still in complex with SGEF we performed a series of immunoprecipitation analyses. HeLa cells were immunoprecipitated using either a pre-immune antibody or anti SGEF antibody, and complexed hDlg was detected using anti-hDlg-1 antibodies. The results in Figure 7A show clear interaction between hDlg and SGEF in HeLa cells. This raised the possibility that E6 might also be present in this complex, so the immunoprecipitates were also analysed for the presence of HPV-18 E6, and, as can be seen from Figure 7A, co-immunoprecipitation of E6 with hDlg and SGEF was also observed. Similarly, when hDlg-1 was immunoprecipitated, both SGEF and HPV-18 E6 could also be detected in the co-immunoprecipitating complexes (Figure S3). We also examined whether hDlg and SGEF could be found in a complex in other HPV transformed cells. To do this hDlg was immunoprecipitated from HPV positive cervical tumour derived Me180 and CaSki cells, as well as from the HPV-16 non-tumourigenic immortalized W12 cells, and complexed SGEF was detected by western blotting. The results in Figure 7B also confirm the interaction between Dlg and SGEF in these other HPV positive cell lines, although the interaction was harder to detect in the W12 cell line.

Since the interaction between E6 and Dlg involves PDZ domain recognition, in a manner similar to that linking Dlg and SGEF, we wanted to determine whether the ability of E6 to complex with Dlg and SGEF was also dependent upon its PBM. To do this cells were transfected with Dlg and SGEF expression constructs, and cell extracts were used in GST pull -down assays with wild type HPV-18 E6 and HPV-18 E6 mutated in the PBM. Bound proteins were detected by western blotting and the results obtained are shown in Figure 7C. As can be seen, the interaction between E6 and Dlg is strictly PDZ domain-dependent, in agreement with previous studies [16]. Most importantly however, SGEF is also pulled down by 18E6, but only when Dlg is present and in a PBM dependent manner, although the GST. ΔPDZ18E6 is expressed at a slightly lower level. These results provide further evidence for the existence of a complex involving Dlg, SGEF and HPV-18 E6.

**Figure 8:**
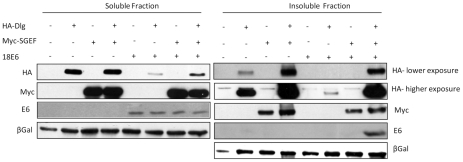
HPV18E6 does not degrade the NP-40 insoluble pool of hDlg in the presence of SGEF. HEK293 cells were transfected with HA-tagged Dlg, Myc-tagged SGEF, untagged HPV18E6 expression plasmids either alone or in different combinations as indicated. After 24 hrs the cells were extracted into NP-40 soluble and NP-40 insoluble fractions and the patterns of expressions of Dlg, SGEF and E6 determined by western blot analysis. β-gal expression was used as a marker for transfection efficiency and a loading control.

To investigate this further we performed a series of transient overexpression experiments in 293 cells. The cells were transfected with an untagged E6 expression vector, together with Dlg and SGEF. Cell extracts were divided into NP-40 soluble and insoluble pools and the levels of expression of E6, Dlg and SGEF were analysed by western blotting. The results in Figure 8 demonstrate a number of interesting features. Co-expression of Dlg and SGEF results in a marked accumulation of both proteins in the NP-40 insoluble compartment. When Dlg and E6 are co-transfected, E6 induces a dramatic decrease in the levels of Dlg, particularly in the NP-40 soluble fraction of the cells. However, the levels of Dlg are largely unaffected by E6 in the NP-40 insoluble pool when SGEF is also present. Furthermore, E6 also becomes localized to this NP-40 insoluble fraction when both SGEF and Dlg are present. Taken together, these results demonstrate that E6, Dlg and SGEF can exist in complex within the NP-40 insoluble fraction of the cell, and that this is dependent upon the ability of E6 to bind Dlg in a PDZ-domain dependent manner.

To determine whether E6 overexpression could influence the pattern of SGEF expression, we co-transfected HA-tagged HPV-18 E6 and Flag-tagged SGEF into HaCaT epithelial cells, and after 24 hrs analysed their respective patterns of expression by immunofluorescence and confocal microscopy. The results in Figure S4 show that SGEF by itself has a largely nuclear pattern of expression, similar to that seen in Figure 4B. However, in the presence of ectopically expressed E6 there is a marked accumulation of SGEF within the cytoplasmic portion of the cell, suggesting that E6 can affect the pattern of SGEF localization.

### hDlg and SGEF both contribute to the invasive capacity of HPV transformed cells

**Figure 9:**
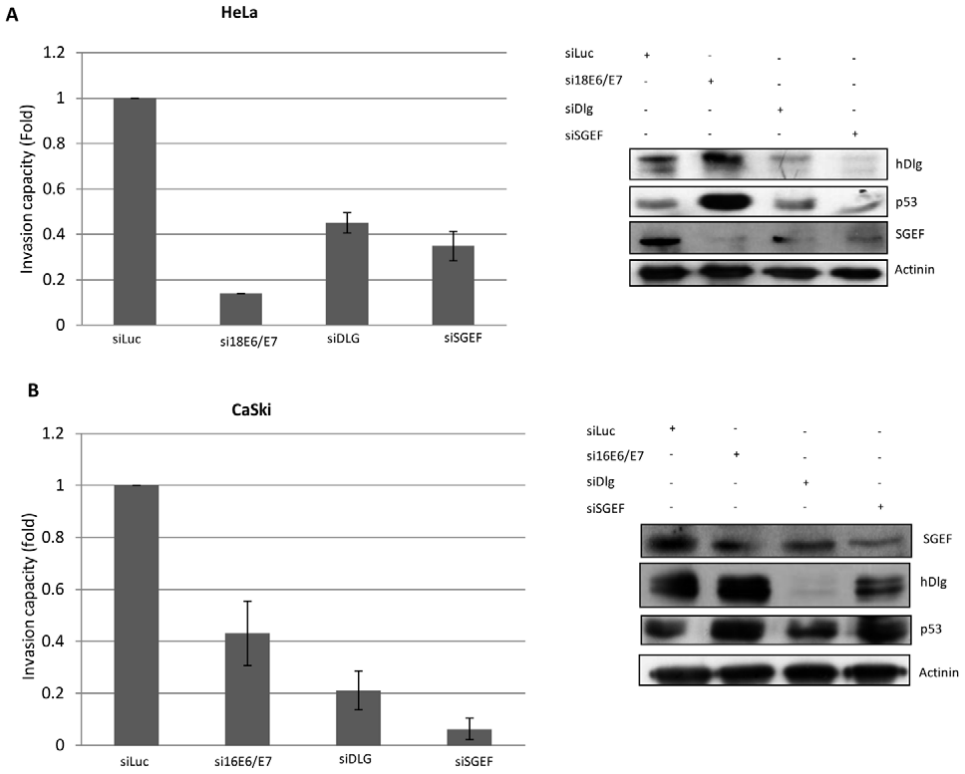
The hDlg/SGEF module is required for the invasive capacity of HeLa and CaSki cells. The cells (HeLa in Panel A and CaSki in Panel B) were transfected with siRNAs to Luciferase (Luc), E6/E7, hDlg or SGEF and after 72 hrs the cells were harvested and equal numbers plated onto Matrigel invasion chambers. After overnight incubation the numbers of invading cells in the lower chamber were counted. The graphs show the fold change in the numbers of invading cells from multiple assays, where siLuc- transfected cells were scored as the reference point. Error bars represent ±SD of multiple experiments. The right hand panels show the western blot analysis of the levels of expression in total cell extracts of hDlg, p53 and SGEF following siRNA transfections performed in parallel with the invasion assays. α-Actinin is shown as the loading control.

HeLa cells are highly tumourigenic and exhibit invasive capacity in matrigel assays [49]. Based upon the above results, we reasoned that this invasive capacity might be dependent upon the active Dlg/SGEF pool that is maintained by E6/E7 expression. To investigate this we proceeded to perform a series of matrigel invasion assays using HeLa cells in which either E6/E7, hDlg or SGEF expression was ablated by siRNA transfection. Cells were transfected with the relevant siRNAs and after 72 hrs the cells were trypsinised and counted, and equal numbers of cells were inoculated into matrigel invasion chambers. At the same time, the efficiency of the siRNAs was determined by western blotting for the relevant target protein (Figure 9A). Chambers were left overnight at 37°C and the number of cells invading the lower chamber was counted the following day. The collated results from multiple assays are also shown in Figure 9A. Not surprisingly, loss of E6/E7 expression results in a dramatic decrease in the invasive capacity of these cells. However, loss of either hDlg or SGEF also results in a significant decrease in the capacity of these cells to invade the matrigel. We also repeated this assay in HPV-16 positive CaSki cells and the results shown in Figure 9B also confirm that continued expression of hDlg and SGEF contribute directly to the invasive potential of these cells. These results demonstrate that, in the context of HPV-induced malignancy, the residual levels of hDlg found within HPV-18 positive HeLa cells and HPV-16 positive CaSki cells actually contribute directly to the invasive potential of these cells, most likely through up-regulation of SGEF and RhoG activity.

Finally we wanted to determine whether Dlg and SGEF could also contribute to the invasive potential of other non-HPV containing tumour derived cells. To do this we analysed the effects of siRNA induced ablation of hDlg-1 and SGEF upon the invasive capacity of non-small cell lung cancer derived H1299 cells. The results obtained are shown in Figure S5 and demonstrate that both hDlg and SGEF also contribute towards the invasive potential of these cells. This suggests that both SGEF and Dlg might be involved in the invasion of diverse tumour types. However, in the context of HPV induced malignancy, where a subset of Dlg is normally degraded by E6, the virus maintains this invasive potential by increasing the levels of SGEF expression and by preserving this SGEF bound pool of Dlg.

## Discussion

hDlg was the first PDZ domain-containing substrate of HPV-18 E6 to be identified, and a number of studies have shown that one of the consequences of this interaction is the proteasome-mediated degradation of hDlg. However E6-mediated degradation of hDlg is not complete, and significant levels of hDlg expression remain in cervical tumour-derived cell lines, raising questions about the role of hDlg in HPV-induced malignancy. As a means of understanding hDlg function more fully, and by analogy its role in HPV-induced pathogenesis, we used a proteomic approach to look for novel interacting partners of hDlg. In this analysis we have characterised one of these hDlg interactions; that with the RhoG specific exchange factor, SGEF, and determined the relevance of this association for the regulation of RhoG activity and for HPV-induced malignancy.

SGEF was initially found in a screen for androgen-responsive genes in human prostate cancer cells [50]. It contains an amino-terminal proline-rich region (Pro), a Dbl homology (DH) domain followed by a Pleckstrin homology (PH) domain, an SH3 domain and a putative PBM [50]. There are several studies reporting the interaction between PDZ domain- containing proteins of the MAGUK family and cellular GEFs, and in most cases, the interaction is mediated through PDZ domain recognition [51], [29], [52], [53]. For instance, Net1, a RhoA specific exchange factor, interacts through its PBM with the tumour suppressor proteins of the Dlg family, including hDlg/SAP97, SAP102, and PSD95 [29]. Here we provide evidence that hDlg and SGEF can interact in a number of different cell types. Mutational analyses demonstrate that the interaction between hDlg and SGEF occurs through two distinct interaction sites and involves both PDZ domain and SH3 domain recognition, although the PBM-PDZ domain interaction appears to be the stronger of the two. The precise stoichiometry of the interaction remains to be determined, as at present it is unclear whether two molecules of SGEF can bind hDlg simultaneously, or whether a single molecule of SGEF binds to hDlg through two separate domains.

From a physiological point of view, the PDZ-PBM interaction between hDlg and SGEF seems to be the most important, since the ability of hDlg to affect the pattern of SGEF expression is PDZ domain-dependent and does not seem to be influenced by SH3 domain recognition. A number of previous studies have proposed that hDlg can function as a molecular scaffold for many of its target proteins. This would appear to hold true in part with respect to SGEF. Thus, overexpressed Dlg induces a marked relocalisation of SGEF from the nuclear/cytosolic compartments into an insoluble cytoplasmic cytoskeletal compartment. These studies were also supported by a series of ablation experiments where loss of hDlg results in a decrease in the levels of SGEF expression within the insoluble cytoskeletal fraction. Taken together, these studies demonstrate that hDlg regulates SGEF localization through the PDZ-PBM interaction.

SGEF activates RhoG efficiently in vitro and in vivo [45] and this activity appears to be highly dependent upon the levels and localization of hDlg. Thus, whilst overexpressed SGEF can activate endogenous RhoG, in agreement with previous studies [45], addition of Dlg greatly augments this activity of SGEF. The positive influence of hDlg on SGEF activity was further confirmed in HaCaT epithelial cells where stable knock-down of hDlg expression also down-regulated the activation of endogenous RhoG; an effect that was reversed upon re-introducing rat Dlg. These results provide a clear explanation for previously reported studies implicating hDlg in the control of cell migration, with the SGEF interaction being a prime candidate for the molecular mechanism by which hDlg regulates this process.

We then proceeded to investigate the implications of the hDlg/SGEF regulatory complex for the development of cervical malignancy, where the HPV E6 oncoprotein has previously been shown to target hDlg for degradation [14], [54], [16]. In order to determine whether E6 could have any influence on the activity of the hDlg/SGEF/RhoG signaling network, we made use of HPV-18 containing HeLa cells that are derived from a cervical tumour. Not surprisingly, siRNA ablation of SGEF expression greatly reduces the levels of RhoG activity in these cells, demonstrating that active RhoG in HeLa is SGEF dependent. Surprisingly, silencing of HPV18E6/E7 expression also down-regulates the levels of RhoG activation. This seems somewhat paradoxical, since hDlg levels show a significant increase upon removal of E6 expression. In order to determine whether the residual hDlg in HeLa cells was in any way influencing SGEF activity, we performed transient siRNA to hDlg and also found a striking decrease in the levels of RhoG activity, similar to that seen with the removal of E6/E7 or SGEF expression. These results demonstrate that high levels of RhoG activity in HeLa are dependent upon SGEF, hDlg and E6/E7 expression. This also suggests that the residual hDlg in HeLa cells that is not degraded by E6 can influence RhoG activity, and also implies that this pool of hDlg is either unaffected by E6 or is actively maintained by the viral oncoproteins. To investigate this we performed cell fractionation assays following removal of E6/E7 expression, and found stabilization of hDlg only within the NP-40 soluble fraction of the cell. Interestingly, there was a significant concomitant decrease in the levels of hDlg in the NP-40 insoluble pool and this was accompanied by an equivalent decrease in SGEF levels. Similar results were also obtained in HPV-16 positive CaSki cells, where we found that the presence of the NP-40 insoluble pool of SGEF was also dependent upon continued E6/E7 expression. These results indicate that whereas NP-40 soluble pools of hDlg are degraded by E6, the insoluble cytoskeletal-bound forms of hDlg/SGEF are actually maintained by the presence of the HPV oncoproteins. This was also confirmed in transient overexpression experiments where E6 actively degrades NP-40 soluble forms of Dlg, but has minimal effect upon the NP-40 insoluble forms when SGEF is also overexpressed; interestingly E6 would also appear to be present in this NP-40 insoluble compartment under these conditions. These results imply that E6 may exist in complex with the Dlg and SGEF, and indeed, co-immunoprecipitation analyses in HeLa cells support this. To gain insight into how this complex might be formed we performed pull-down assays using wild type and a ΔPBM mutant of E6. In these assays we could clearly see that E6 could pull down SGEF from cell extracts only in the presence of Dlg, and only if it had an intact PBM. Taken together, these results indicate that E6 can exist in complex with both hDlg and SGEF in HPV-18 transformed cells and that this complex is responsible for the high levels of RhoG activity found in these cells.

During the course of these studies we also noticed that loss of E6/E7 expression has a generally deleterious effect upon the levels of SGEF expression. Using different targeting siRNAs we found that loss of E6 alone has minimal effect upon total SGEF levels of expression, whilst loss of both E6 and E7 results in a marked decrease in the levels of SGEF expression. This suggests that E7 may contribute directly to maintaining high levels of SGEF expression in these HPV transformed cells. Indeed, SGEF is subject to regulation by E2F-1 [47], suggesting that one potential means by which E7 could achieve this is via degradation of pRb and increased levels of E2F-1 activity [8], [48]. These studies also provide a further demonstration of the cooperativity in function between E6 and E7. In this case, E7 contributes by maintaining high levels of SGEF expression, whilst E6 contributes by retaining a pool of Dlg/SGEF favourable for maintaining high levels of RhoG activity.

Finally, we wanted to determine whether this insoluble cytoskeletal bound pool of hDlg and SGEF was biologically relevant in the context of the invasive capacity of HPV transformed cells. Using matrigel invasion assays we found that invasion was critically dependent upon the continued expression of E6 and E7 in HPV-18 and HPV-16 positive tumour-derived cell lines. Importantly, we also found that both hDlg and SGEF play vital roles and are essential for the optimal invasive potential of these cells. This is consistent with previous studies showing that RhoG is also required for cell migration and invasion of HeLa cells [55] and breast cancer cells [56], and also with our data showing that the invasive potential of a HPV negative lung cancer-derived cell line is also dependent upon Dlg and SGEF expression. Taken together, these studies demonstrate that the SGEF/hDlg/RhoG module can directly control a tumour cell's invasive potential. Based on this we can propose a model where hDlg recruits SGEF to the cytoskeleton, resulting in increased levels of RhoG activation and increased levels of invasion. In the context of HPV-induced malignancy a significant amount of hDlg is degraded by E6, however this cytoskeletal bound form of hDlg in complex with SGEF is actively maintained by the combined action of E6 and E7, thereby maintaining the invasive potential of the cells. Whilst it would be naïve to assume that this is the only activity required for the invasiveness of HPV transformed cells, it nonetheless demonstrates that loss of either hDlg or SGEF alone is sufficient to abolish their invasive capacity.

At present we have no information on what the role of the E6-Dlg interaction might have with respect to the viral life cycle, although loss of PDZ binding capacity in the context of the viral genome is highly deleterious [57], [58]. It is hard to envisage how effects on cell invasive potential would be of relevance to the viral life cycle, and thus this particular readout may simply reflect downstream effects of malignancy. However, it is intriguing to note that RhoG activity has been implicated in the control of proliferation in stem cell-like neuronal cells [59] and in differentiation in the context of Trio induced neurite outgrowth [60].Therefore it is possible that in the HPV-infected keratinocyte, activation of the RhoG pathway may also induce cell proliferation with obvious benefits to the viral life cycle. Future studies will be aimed at addressing these issues.

## Materials and Methods

### Cell culture and transfections

HEK293 (Human embryonic kidney) cells, non-small cell lung cancer H1299 cells, HaCaT epithelial cells, HPV-18 positive HeLa, HPV-68b positive Me180 cells and HPV-16 positive CaSki cells were grown in DMEM supplemented with 10% fetal bovine serum, penicillin–streptomycin (100 U/ml) and glutamine (300 µg/ml). HPV-16 positive W12 cells were grown in F12 DMEM supplemented with 5% fetal bovine serum. Cells were transfected using the standard calcium phosphate precipitation or Lipofectamine 2000 (Invitrogen). HaCaT stable Dlg knockdown cells have been described previously [61].

### Plasmid constructs

The HA-tagged wild type and mutant DLG expression constructs have been described previously [62]. The mutant DLG constructs used in this study are as follows: HA-DLG-NTPDZ1-2 refers to aa 1–382; HA-CT refers to aa 539–921. GST-wtDLG refers to aa 1–921; GSTDlg-NT: aa 1–222; GST-DlgNT1: aa 1–276; GSTDlgNT1/2: aa 1–382. The pGEX-HPV18E6, the untagged pCDNA HPV18E6, pCDNA HA-tagged HPV18E6 constructs and the GST-wt18E6 have been described previously [63], [64] and GST-ΔPDZ18E6 construct was generated using Gene Tailor Site directed Mutagenesis System kit (Invitrogen). Flag-tagged wtSGEF was a kind gift from Jayesh C. Patel (Section of Microbial Pathogenesis, Yale University School of Medicine, Boyer Center for Molecular Medicine, New Haven). Myc-tagged constructs of SGEF: wtSGEF, ΔPDZSGEF and ΔSH3SGEF [45] were cloned into pCMVmyc into EcoRI and BamHI sites (Clontech). The GST-ELMO construct was a kind gift from Dr. Keith Burridge (Department of Cell and Developmental Biology, University of North Carolina, USA).

### Antibodies

Primary antibodies used were mouse anti-HA monoclonal antibody (Roche), mouse anti-Myc (Santa Cruz), rabbit anti-SGEF (Sigma), mouse anti-RhoG (Millipore), mouse monoclonal anti-α-tubulin (Sigma); mouse monoclonal anti-p53 (DO-1), rabbit polyclonal anti-α-actinin (Santa Cruz), mouse monoclonal anti-Dlg (Santa Cruz) mouse monoclonal anti-p84 (Abcam) mouse anti-β-galactosidase monoclonal antibody (Promega), mouse monoclonal anti-HPV18E6 (Arbor Vita Corporation), mouse anti-vimentin monoclonal antibody (Santa Cruz) and the secondary anti-mouse and anti-rabbit antibodies were conjugated to horseradish peroxidase (Dako). The proteins were visualized by enhanced chemiluminesence (GE Healthcare) according to the manufacturer's instructions.

### Western Blot analysis

For total cellular lysate preparation, cells were lysed in 2× sodium dodecyl sulfate (SDS) sample buffer (100 mM Tris HCl [pH 6.8], 200 mM dithiothreitol [DTT], 4% SDS, 20% glycerol, 0.2% bromophenol blue) and for the soluble and insoluble cellular lysate preparation, cells were lysed with a extraction buffer (E1A) containing 25 mM HEPES pH 7.0, 0.1% NP-40, 150 mM NaCl, protease inhibitor cocktail I (Calbiochem). The extracts were allowed to stand on ice for 30 min, following which the lysates were clarified by centrifugation. The supernatant was collected in fresh tubes as the NP-40 soluble cellular fraction whilst the pellet was resuspended in SDS PAGE sample buffer and sonicated briefly; this was used as the NP-40 insoluble fraction. Cell lysates, typically 50 µg, were subjected to SDS-PAGE and then electrophoretically transferred to nitrocellulose membranes (Schleicher and Schuell). The membranes were blocked at 37°C for 1 h in 10% milk-phosphate-buffered saline (PBS), except for those probed with anti-SGEF and anti-RhoG antibodies, which were blocked in 5% milk-TBS with 0.1% Tween20. The blots were incubated with the appropriate primary antibodies diluted in the washing buffer (10% milk-PBS, 0.5% Tween 20), except for the anti-SGEF and anti-RhoG antibodies, which were diluted in 5% milk-TBS and 0.1% Tween 20. The incubation times were 2 h at room temperature for all antibodies, except for the anti-SGEF, anti-RhoG and anti-E-cadherin, which were incubated overnight at 4°C. After several washes, the membranes were incubated with the appropriate horseradish peroxidase (HRP)-conjugated secondary antibody (Dako) for 1 h at room temperature. After extensive washing, the blots were developed with the enhanced chemiluminescence (ECL) or ECL Plus reagent (GE Healthcare) according to the manufacturer's instructions. Protein band intensities in the case of GST-ELMO pull down assays were quantitated using ImageJ software.

### Immunoprecipitation and mass spectrometry analysis

HEK293 cells were transfected with the appropriate plasmids. After 24 h, cells were extracted in mass spectrometry lysis buffer (50 mM HEPES, pH 7.4 [at 4°C], 150 mM NaCl, 50 mM NaF, 1 mM EDTA, 0.25% NP-40), and extracts were incubated with anti-HA beads (Sigma) for 2 to 3 h on a rotating wheel at 4°C. The beads were then extensively washed, dried, and subjected to proteomic analysis as described previously [64].

### Peptide pull-down and GST pull-down assays

Peptide pull-down assays were performed as previously described [29]. HEK293 cells were seeded in 10 cm^2^ dishes, the following day the cells were transfected with relevant plasmids. After 24 hrs, cellular extracts were prepared using E1A buffer. The different samples were equalized for the protein concentration and volume, then incubated with GST fusion proteins immobilized on glutathione agarose for approximately 3–4 hrs at 4°C on a rotating wheel. After extensive washing, the bound proteins were detected using SDS-PAGE and Western blotting.

### Rho GTPase activation assays

The amount of activated, GTP-bound Rho proteins was measured as described previously [45]. Briefly, pull-down assays were done using purified GST-ELMO (GST fusion protein containing the full-length RhoG effector, ELMO) with cell extracts from HEK293 cells transfected with vector control, Dlg and SGEF either alone or in combination. Similar pull-down assays were performed with HeLa cells transfected with relevant siRNAs. Cellular extracts were prepared from HEK293 cells and HeLa cells after 24 h or 72 h respectively by lysing the cells in 250 µl of 50 mM Tris, pH 7.4, 10 mM MgCl2, 500 mM NaCl, 1% Triton X-100, 0.1% SDS, 0.5% deoxycholate, and protease inhibitors and then equalized for protein concentration. Lysates (700–800 µg) were cleared at 16,000× g for 5 min. Supernatants were incubated with purified GST-ELMO conjugated to glutathione-Sepharose beads for 20 mins at 4°C. After extensive washing the bound RhoG was analysed by western blotting and compared with the total RhoG present within the cell lysate.

### Immunofluorescence

HaCaT cells were seeded at low cell density on glass coverslips and were transfected with relevant plasmid DNA using calcium phosphate precipitation. 24 hrs post transfection, cells were washed in PBS and fixed in 3.7% paraformaldehyde in PBS followed by 5 min in 0.1% Triton-PBS. The cells were then stained with anti-HA monoclonal antibody (Roche) or anti-Flag monoclonal antibody (Sigma) for 2 h at 37°C, washed extensively in PBS, and incubated for 20 min at 37°C with a secondary anti-rabbit or anti-mouse antibody conjugated to fluorescein or rhodamine (Molecular Probes). Samples were washed several times with PBS and were mounted with Vectashield mounting medium (Vector Laboratories) on glass slides and visualized using a Zeiss LSM 510 confocal microscope.

### siRNA experiments

For transient siRNA experiments, cells were seeded in 6-cm^2^ dishes and transfected using Lipofectamine 2000 (Invitrogen) with the following siRNAs from Dharmacon: siRNA against luciferase as a control, siRNA against HPV-18 E6/E7 (CAUUUACCAGCCCGACGAG), siRNA against HPV-18 E6 (CUAACUAACACUGGGUUAU), siRNA against HPV-16 E6/E7 (UUAAAUGACAGCUCAGAGG), siRNA against hDlg and siRNA against SGEF.

### Subcellular fractionation assays

HEK293 cells were transfected with the relevant plasmids. After 24 hrs, differential extraction of HEK293 cells was performed to obtain cytoplasmic, membrane, nuclear and cytoskeletal fractions using the ProteoExtract Fractionation Kit (Calbiochem) according to the manufacturer's instructions.

### Co-immunoprecipitations

Cells were seeded in 10-cm^2^ dishes. After 24 hrs, cell extracts were prepared in E1A buffer containing protease inhibitor. The extracts were then passed through a 26G needle multiple times and then cleared by centrifugation. An equal concentration of protein from the cellular extracts was incubated with either the SGEF antibody or the control antibody for approximately 3–4 hrs on a rotating wheel at 4°C. Protein-A-Sepharose beads (GE Healthcare) were then added for an additional 50 minutes at 4°C. The beads were washed three times with extraction buffer containing protease inhibitor and precipitated proteins were analysed by Western blot.

### Matrigel invasion assays

The matrigel invasion assay was performed using BD Matrigel Invasion chambers (BD BioCoat) as per the company's instructions. In brief, 1.5×10^5^ cells were seeded in 6-cm^2^ dishes and the next day were transfected using Lipofectamine 2000 (Invitrogen) with relevant siRNA. After 72 hrs, cells were trypsinised and counted. Equal numbers of cells were seeded in serum free medium into the wells and medium containing 20% serum was added to the lower chambers as a chemo attractant. After 20 hrs, the cells that had invaded through the matrigel and were on the lower surface of the chamber were stained using crystal violet. The entire number of cells that migrated in each assay (performed in duplicate) were then counted.

## Supporting Information

Figure S1 **Dlg recruits SGEF to the cytoplasmic cytoskeletal network.** HaCaT cells transfected with HA-Dlg, Flag-SGEF or co-transfected with the two cDNAs were fixed and processed for immunofluorescence with anti-Flag to detect SGEF and anti-HA to detect Dlg. The upper six panels show typical staining patterns for SGEF and Dlg when transfected alone. The lower nine panels show the distribution patterns when Dlg and SGEF are co-transfected.
(TIF)

Figure S2 **HPV-16 E6/E7 are required for maintaining SGEF expression in CaSki cells.** HPV-16 containing CaSki cells were transfected with control siRNA (luc) or siRNA to 16E6/E7 and analysed for the levels of SGEF and p53 expression in the NP-40 soluble and insoluble fractions of the cell after 72 hrs. α-Actinin was used as a loading control.
(TIF)

Figure S3 **HPV-18E6, hDlg and SGEF exist in a complex.** HeLa cells were seeded in 10 cm^2^ dishes. After 24 hrs, cellular extracts were prepared from these cells and immunoprecipitated using either the control antibody or the anti-hDlg-1 antibody. SGEF and HPV18-E6 bound to the Dlg were detected using the anti-SGEF and anti-E6 antibodies respectively. The immunoprecipitated Dlg was detected using anti-hDlg-1 antibody. The bottom 3 lanes show the input levels for hDlg, HPV18-E6 and SGEF used in this assay.
(TIF)

Figure S4 **HPV-18E6 can influence the pattern of SGEF expression.** HaCaT cells were transfected with Flag-tagged SGEF and HA-tagged HPV-18E6, either alone or in combination. After 24 hrs the cells were fixed and processed for immunofluorescence with anti-Flag and anti-HA antibodies. The upper two panels show the pattern of expression of SGEF and E6 alone, whilst the lower panels shows the patterns of SGEF expression in two cells with high and low levels of E6 expression.
(TIFF)

Figure S5 **Invasive potential of H1299 cells is dependent upon hDlg and SGEF.** H1299 cells were transfected with siRNAs to Luciferase (Luc), hDlg or SGEF and after 72 hrs the cells were harvested and equal numbers plated onto Matrigel invasion chambers. After overnight incubation the numbers of invading cells in the lower chamber were counted. The graph shows the fold change in the numbers of invading cells from multiple assays, where siLuc- transfected cells were scored as the reference point. Error bars represent ±SD of multiple experiments. The lower panel shows the western blot analysis of the levels of expression in total cell extracts of hDlg and SGEF following siRNA transfections performed in parallel with the invasion assays. α-Actinin is shown as the loading control.
(TIF)

## References

[1] zur Hausen H (1999). Immortalization of human cells and their malignant conversion by high-risk human papillomavirus genotypes.. Semin Cancer Biol.

[2] zur Hausen H (2002). Papillomaviruses and cancer: from basic studies to clinical application.. Nat Rev Cancer.

[3] Barbosa MS, Schlegel R (1989). The E6 and E7 genes of HPV-18 are sufficient for inducing two-stage in vitro transformation of human keratinocytes.. Oncogene.

[4] Hawley-Nelson P, Vousden KH, Hubbert NL, Lowy DR, Schiller JT (1989). HPV-16 E6 and E7 proteins cooperate to immortalise human foreskin keratinocytes.. EMBO J.

[5] Song S, Liem A, Miller JA, Lambert PF (2000). Human papillomavirus type 16 E6 and E7 contribute differently to carcinogenesis.. Virology.

[6] zur Hausen H, Rigby PW, Wilkie NM (1986). Genital papillomavirus infections.. Viruses and cancer.

[7] de Villiers EM, Fauquet C, Broker TR, Bernard HU, zur Hausen H (2004). Classification of papillomaviruses.. Virology.

[8] Dyson N, Howley PM, Munger K, Harlow E (1989). The human papilloma virus-16 E7 oncoprotein is able to bind to the retinoblastoma gene product.. Science.

[9] Boyer SN, Wazer DE, Band V (1996). E7 protein of human papilloma virus-16 induces degradation of retinoblastoma protein through the ubiquitin-proteasome pathway.. Cancer Res.

[10] Werness BA, Levine AJ, Howley PM (1990). Association of human papillomavirus types 16 and 18 E6 proteins with p53.. Science.

[11] Münger K, Basile JR, Duensing S, Eichten A, Gonzalez SL (2001). Biological activities and molecular targets of the human papillomavirus E7 oncoprotein.. Oncogene.

[12] Mantovani F, Banks L (2001). The human papillomavirus E6 protein and its contribution to malignant progression.. Oncogene.

[13] Thomas M, Narayan N, Pim D, Tomaić V, Massimi P (2008). Human papillomaviruses, cervical cancer and cell polarity.. Oncogene.

[14] Kiyono T, Hiraiwa A, Fujita M, Hayashi Y, Akiyama T (1997). Binding of high-risk human papillomavirus E6 oncoproteins to the human homologue of the Drosophila discs large tumor suppressor protein.. Proc Natl Acad Sci U S A.

[15] Javier RT (2008). Cell polarity proteins: common targets for tumorigenic human viruses.. Oncogene.

[16] Gardiol D, Kühne C, Glaunsinger B, Lee SS, Javier R (1999). Oncogenic human papillomavirus E6 proteins target the discs large tumour suppressor for proteasome-mediated degradation.. Oncogene.

[17] Woods DF, Bryant PJ (1991). The discs-large tumor suppressor gene of Drosophila encodes a guanylate kinase homolog localized at septate junctions.. Cell.

[18] Woods DF, Bryant PJ (1993). ZO-1, Dlg-A and PSD-95/SAP90 homologous proteins in tight, septate and synaptic cell junctions.. Mech Dev.

[19] Woods DF, Hough C, Peel D, Callaini G, Bryant PJ (1996). Dlg protein is required for junction structure, cell polarity, and proliferation control in Drosophila epithelia.. J Cell Biol.

[20] Budnik V, Koh YH, Guan B, Hartmann B, Hough C (1996). Regulation of synapse structure and function by the Drosophila tumor suppressor gene dlg.. Neuron.

[21] Garner CC, Kindler S (1996). Synaptic proteins and the assembly of synaptic junctions.. Trends Cell Biol.

[22] Howard MA, Elias GM, Elias LA, Swat W, Nicoll RA (2010). The role of SAP97 in synaptic glutamate receptor dynamics.. Proc Natl Acad Sci U S A.

[23] Lue R, Marfatia S, Branton D, Chishti A (1994). Cloning and characterisation of hDlg: the human homologue of the Drosophila discs large tumour suppressor binds to protein 4.1.. Proc Natl Acad Sci U S A.

[24] Lue RA, Brandin E, Chan EP, Branton D (1996). Two independent domains of hDlg are sufficient for subcellular targeting: the PDZ1-2 conformational unit and an alternatively spliced domain.. J Cell Biol.

[25] Marfatia SM, Lue RA, Branton D, Chishti AH (1994). In vitro binding studies suggest a membrane-associated complex between erythroid p55, protein 4.1, and glycophorin C. J Biol Chem.

[26] Marfatia SM, Morais Cabral JH, Lin L, Hough C, Bryant PJ (1996). Modular organization of the PDZ domains in the human discs-large protein suggests a mechanism for coupling PDZ domain-binding proteins to ATP and the membrane cytoskeleton.. J Cell Biol.

[27] Paarmann I, Spangenberg O, Lavie A, Konrad M (2002). Formation of complexes between Ca2+.calmodulin and the synapse-associated protein SAP97 requires the SH3 domain-guanylate kinase domain-connecting HOOK region.. J Biol Chem.

[28] Wakabayashi M, Ito T, Mitsushima M, Aizawa S, Ueda K (2003). Interaction of lp-dlg/KIAA0583, a membrane-associated guanylate kinase family protein, with vinexin and beta-catenin at sites of cell-cell contact.. J Biol Chem.

[29] García-Mata R, Dubash AD, Sharek L, Carr HS, Frost JA (2007). The nuclear RhoA exchange factor Net1 interacts with proteins of the Dlg family, affects their localization, and influences their tumor suppressor activity.. Mol Cell Biol.

[30] Bilder D, Li M, Perrimon N (2000). Cooperative regulation of cell polarity and growth by Drosophila tumor suppressors.. Science.

[31] Hough CD, Woods DF, Park S, Bryant PJ (1997). Organizing a functional junctional complex requires specific domains of the Drosophila MAGUK Discs large.. Genes Dev.

[32] Caruana G (2002). Genetic studies define MAGUK proteins as regulators of epithelial cell polarity.. Int J Dev Bio.

[33] Frese KK, Latorre IJ, Chung SH, Caruana G, Bernstein A (2006). Oncogenic function for the Dlg1 mammalian homolog of the Drosophila discs-large tumor suppressor.. EMBO J.

[34] Latorre IJ, Roh MH, Frese KK, Weiss RS, Margolis B (2005). Viral oncoprotein-induced mislocalization of select PDZ proteins disrupts tight junctions and causes polarity defects in epithelial cells.. J Cell Sci.

[35] Cavatorta AL, Fumero G, Chouhy D, Aguirre R, Nocito AL (2004). Differential expression of the human homologue of Drosophila discs large oncosuppressor in histologic samples from human Papillomavirus-associated lesions as a marker for progression to malignancy.. Int J Cancer.

[36] Watson RA, Rollason TP, Reynolds GM, Murray PG, Banks L (2002). Changes in expression of the human homologue of the Drosophila discs large tumour suppressor protein in high-grade premalignant cervical neoplasias.. Carcinogenesis.

[37] Iñesta-Vaquera FA, Campbell DG, Arthur JS, Cuenda A (2010). ERK5 pathway regulates the phosphorylation of tumour suppressor hDlg during mitosis.. Biochem Biophys Res Commun.

[38] Narayan N, Massimi P, Banks L (2009). CDK phosphorylation of the discs large tumour suppressor controls its localisation and stability.. J Cell Sci.

[39] Sabio G, Arthur JS, Kuma Y, Peggie M, Carr J (2005). p38gamma regulates the localisation of SAP97 in the cytoskeleton by modulating its interaction with GKAP.. EMBO J.

[40] Massimi P, Narayan N, Cuenda A, Banks L (2006). Phosphorylation of the discs large tumour suppressor protein controls its membrane localisation and enhances its susceptibility to HPV E6-induced degradation.. Oncogene.

[41] Massimi P, Gammoh N, Thomas M, Banks L (2004). HPV E6 specifically targets different cellular pools of its PDZ domain-containing tumour suppressor substrates for proteasome-mediated degradation.. Oncogene.

[42] Lee S, Fan S, Makarova O, Straight S, Margolis B (2002). A novel and conserved protein-protein interaction domain of mammalian Lin-2/CASK binds and recruits SAP97 to the lateral surface of epithelia.. Mol Cell Biol.

[43] van Buul JD, Allingham MJ, Samson T, Meller J, Boulter E (2007). RhoG regulates endothelial apical cup assembly downstream from ICAM1 engagement and is involved in leukocyte trans-endothelial migration. J Cell Biol.

[44] Humbert PO, Grzeschik NA, Brumby AM, Galea R, Elsum I (2008). Control of tumourigenesis by the Scribble/Dlg/Lgl polarity module.. Oncogene.

[45] Ellerbroek SM, Wennerberg K, Arthur WT, Dunty JM, Bowman DR (2004). SGEF, a RhoG guanine nucleotide exchange factor that stimulates macropinocytosis.. Mol Biol Cell.

[46] Katoh H, Negishi M (2003). RhoG activates Rac1 by direct interaction with the Dock180-binding protein Elmo.. Nature.

[47] Iwanaga R, Komori H, Ishida S, Okamura N, Nakayama K (2006). Identification of novel E2F1 target genes regulated in cell cycle-dependent and independent manners.. Oncogene.

[48] Chellappan S, Kraus VB, Kroger B, Munger K, Howley PM (1992). Adenovirus E1A, simian virus 40 tumor antigen, and human papillomavirus E7 protein share the capacity to disrupt the interaction between transcription factor E2F and the retinoblastoma gene product.. Proc Natl Acad Sci U S A.

[49] Chen L, Wu YY, Liu P, Wang J, Wang G (2011). Down-regulation of HPV18 E6, E7, or VEGF expression attenuates malignant biological behavior of human cervical cancer cells.. Med Oncol.

[50] Qi H, Fournier A, Grenier J, Fillion C, Labrie Y (2003). Isolation of the novel human guanine nucleotide exchange factor Src homology 3 domain-containing guanine nucleotide exchange factor (SGEF) and of C-terminal SGEF, an N-terminally truncated form of SGEF, the expression of which is regulated by androgen in prostate cancer cells.. Endocrinology.

[51] Penzes P, Johnson RC, Sattler R, Zhang X, Huganir RL (2001). The neuronal Rho-GEF Kalirin-7 interacts with PDZ domain-containing proteins and regulates dendritic morphogenesis.. Neuron.

[52] Oliver AW, He X, Borthwick K, Donne AJ, Hampson L (2011). The HPV16 E6 binding protein Tip-1 interacts with ARHGEF16, which activates Cdc42.. Br J Cancer.

[53] Shepherd TR, Klaus SM, Liu X, Ramaswamy S, DeMali KA (2010). The Tiam1 PDZ domain couples to Syndecan1 and promotes cell-matrix adhesion.. J Mol Biol.

[54] Lee SS, Weiss RS, Javier RT (1997). Binding of human virus oncoproteins to hDlg/SAP97, a mammalian homolog of the Drosophila discs large tumor suppressor protein.. Proc Natl Acad Sci.

[55] Katoh H, Hiramoto K, Negishi M (2006). Activation of Rac1 by RhoG regulates cell migration.. J Cell Sci.

[56] Hiramoto-Yamaki N, Takeuchi S, Ueda S, Harada K, Fujimoto S (2010). Ephexin4 and ephA2 mediate cell migration through a RhoG-dependent mechanism.. J Cell Biol.

[57] Lee C, Laimins LA (2004). Role of the PDZ domain-binding motif of the oncoprotein E6 in the pathogenesis of human papillomavirus type 31.. J Virol.

[58] Nicolaides L, Davy C, Raj K, Kranjec C, Banks L (2011). Stabilization of HPV16 E6 protein by PDZ proteins, and potential implications for genome maintenance.. Virology.

[59] Fujimoto S, Negishi M, Katoh H (2009). RhoG promotes neural progenitor cell proliferation in mouse cerebral cortex.. Mol Biol Cell.

[60] Estrach S, Schmidt S, Diriong S, Penna A, Blangy A (2002). The Human Rho-GEF trio and its target GTPase RhoG are involved in the NGF pathway, leading to neurite outgrowth.. Curr Biol.

[61] Nagasaka K, Pim D, Massimi P, Thomas M, Tomaić V (2010). The cell polarity regulator hScrib controls ERK activation through a KIM site-dependent interaction.. Oncogene.

[62] Gardiol D, Galizzi S, Banks L (2002). Mutational analysis of the discs large tumour suppressor identifies domains responsible for human papillomavirus type 18 E6-mediated degradation.. J Gen Virol.

[63] Pim D, Massimi P, Banks L (1997). Alternatively spliced HPV-18 E6* protein inhibits E6 mediated degradation of p53 and suppresses transformed cell growth.. Oncogene.

[64] Tomaić V, Gardiol D, Massimi P, Ozbun M (2009). Human and primate tumour viruses use PDZ binding as an evolutionarily conserved mechanism of targeting cell polarity regulators.. Oncogene.

