# Supplementary figures and images for: The Invasive Capacity of HPV Transformed Cells Requires the hDlg-Dependent Enhancement of SGEF/RhoG Activity
Source: PLoS Pathog. 2012 Feb 23;8(2):e1002543. doi: 10.1371/journal.ppat.1002543 (PMC3285591; doi:10.1371/journal.ppat.1002543)

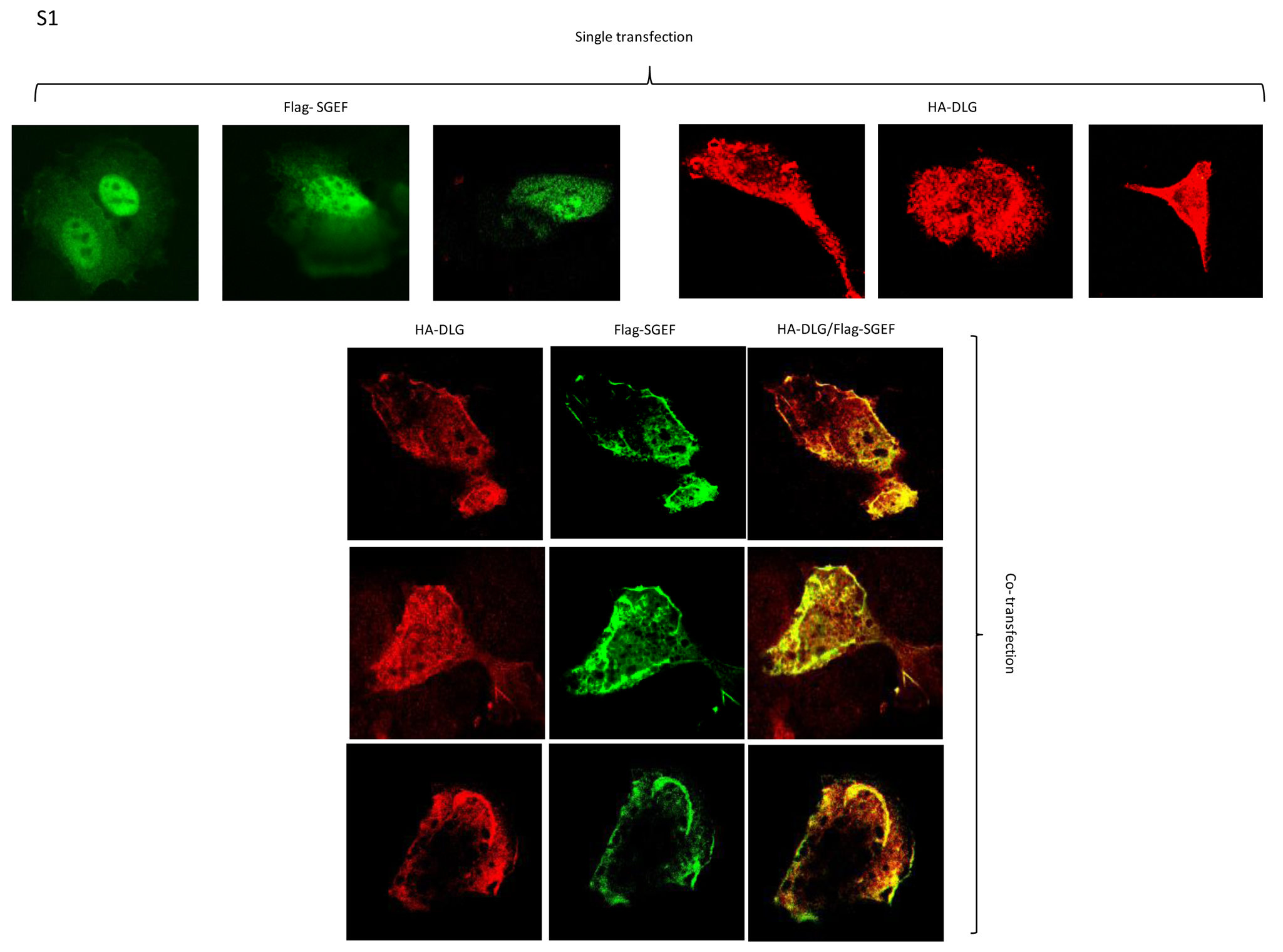

Supplement: Figure S1 — Dlg recruits SGEF to the cytoplasmic cytoskeletal network. HaCaT cells transfected with HA-Dlg, Flag-SGEF or co-transfected with the two cDNAs were fixed and processed for immunofluorescence with anti-Flag to detect SGEF and anti-HA to detect Dlg. The upper six panels show typical staining patterns for SGEF and Dlg when transfected alone. The lower nine panels show the distribution patterns when Dlg and SGEF are co-transfected. (TIF) [file ppat.1002543.s001.tif]

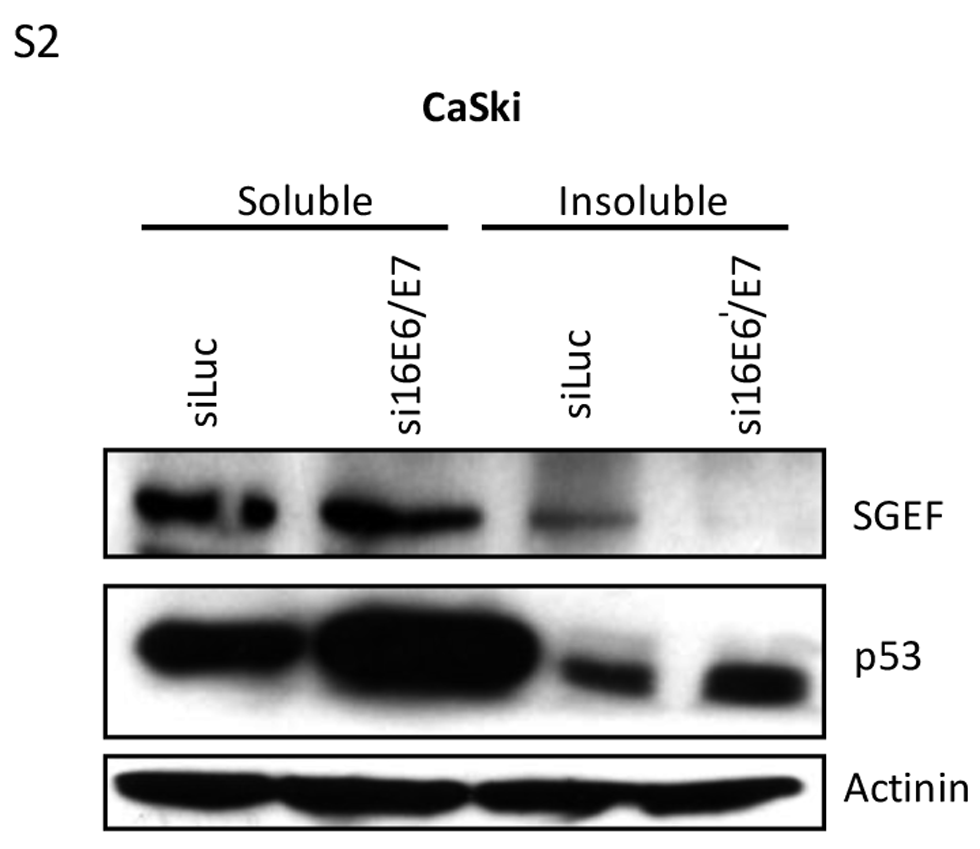

Supplement: Figure S2 — HPV-16 E6/E7 are required for maintaining SGEF expression in CaSki cells. HPV-16 containing CaSki cells were transfected with control siRNA (luc) or siRNA to 16E6/E7 and analysed for the levels of SGEF and p53 expression in the NP-40 soluble and insoluble fractions of the cell after 72 hrs. α-Actinin was used as a loading control. (TIF) [file ppat.1002543.s002.tif]

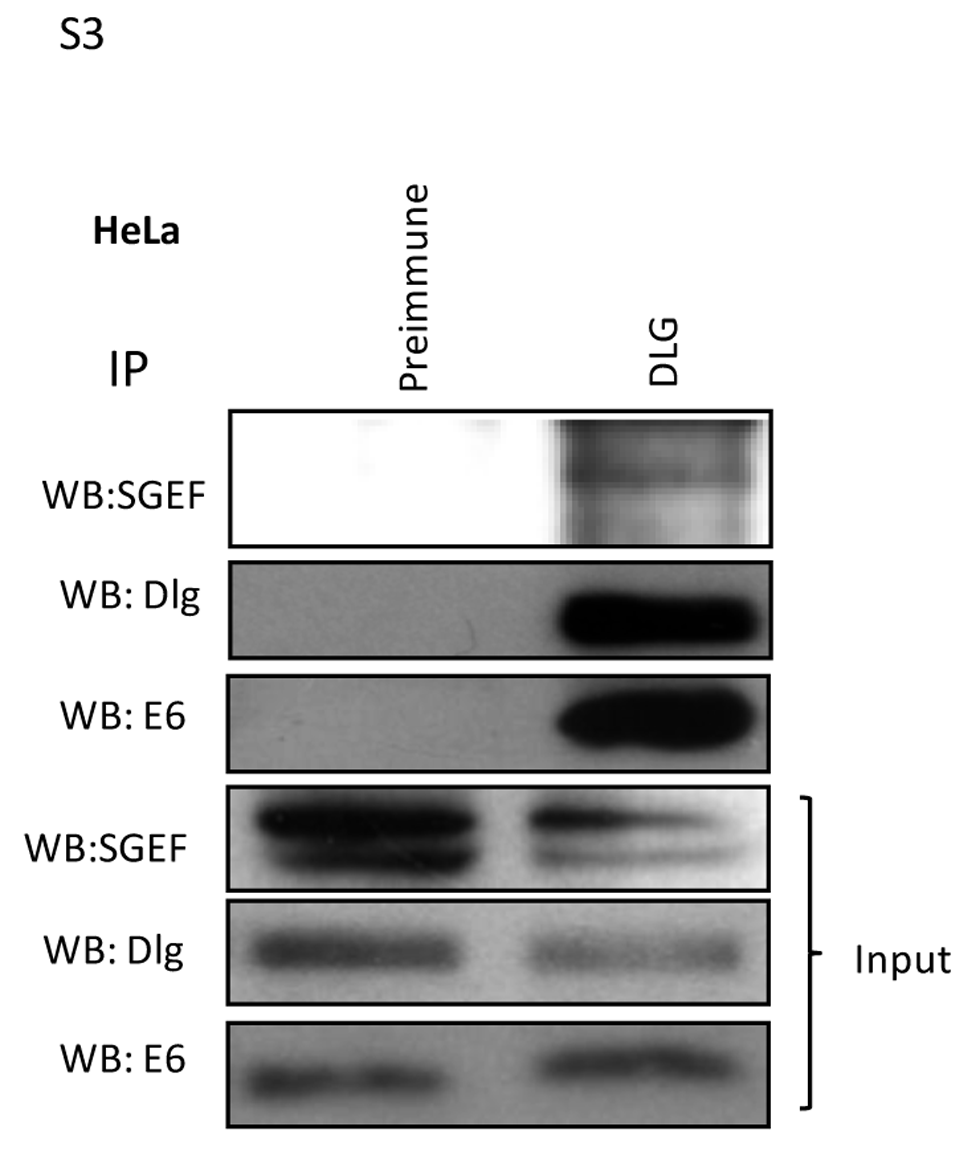

Supplement: Figure S3 — HPV-18E6, hDlg and SGEF exist in a complex. HeLa cells were seeded in 10 cm2 dishes. After 24 hrs, cellular extracts were prepared from these cells and immunoprecipitated using either the control antibody or the anti-hDlg-1 antibody. SGEF and HPV18-E6 bound to the Dlg were detected using the anti-SGEF and anti-E6 antibodies respectively. The immunoprecipitated Dlg was detected using anti-hDlg-1 antibody. The bottom 3 lanes show the input levels for hDlg, HPV18-E6 and SGEF used in this assay. (TIF) [file ppat.1002543.s003.tif]

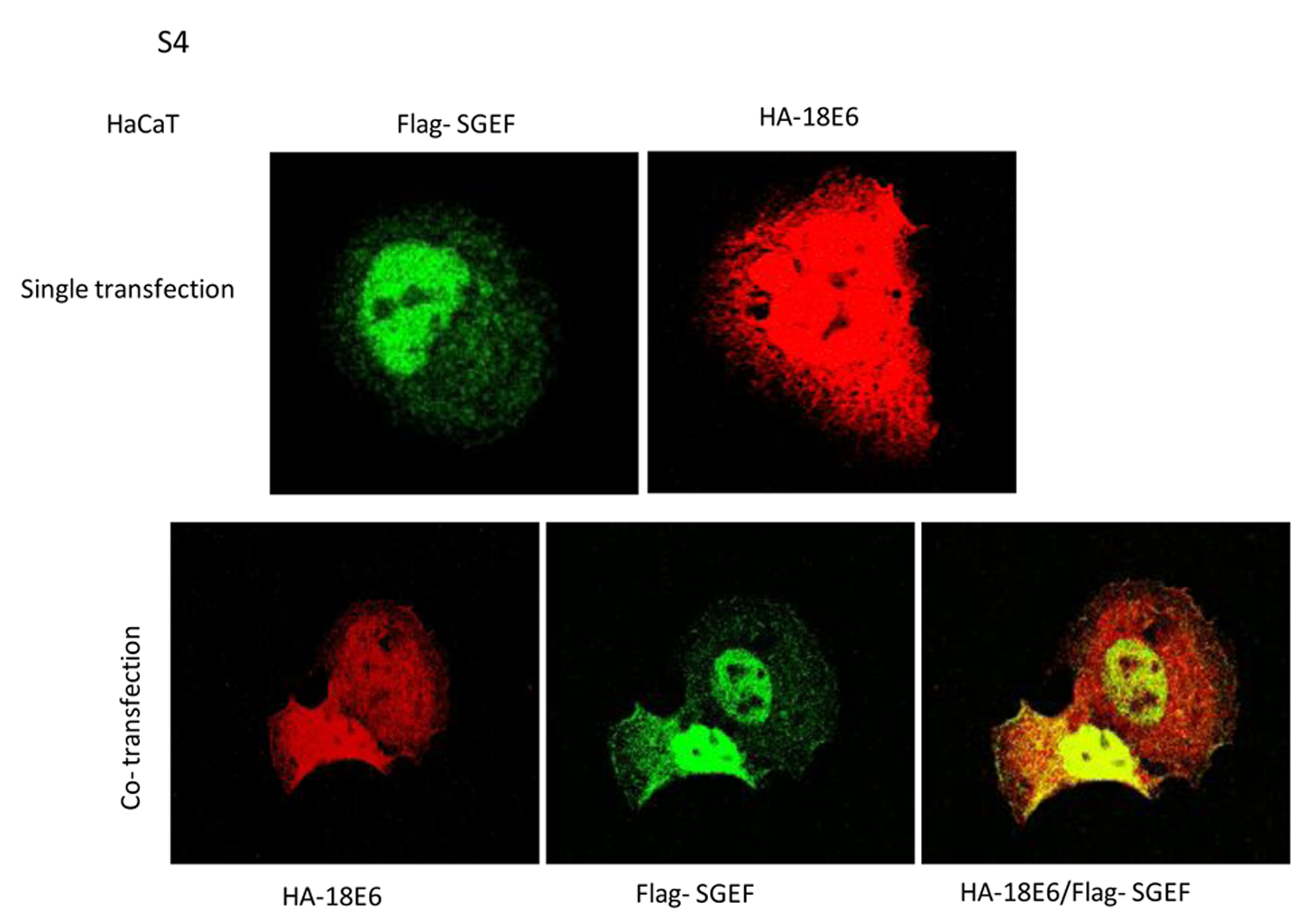

Supplement: Figure S4 — HPV-18E6 can influence the pattern of SGEF expression. HaCaT cells were transfected with Flag-tagged SGEF and HA-tagged HPV-18E6, either alone or in combination. After 24 hrs the cells were fixed and processed for immunofluorescence with anti-Flag and anti-HA antibodies. The upper two panels show the pattern of expression of SGEF and E6 alone, whilst the lower panels shows the patterns of SGEF expression in two cells with high and low levels of E6 expression. (TIFF) [file ppat.1002543.s004.tif]

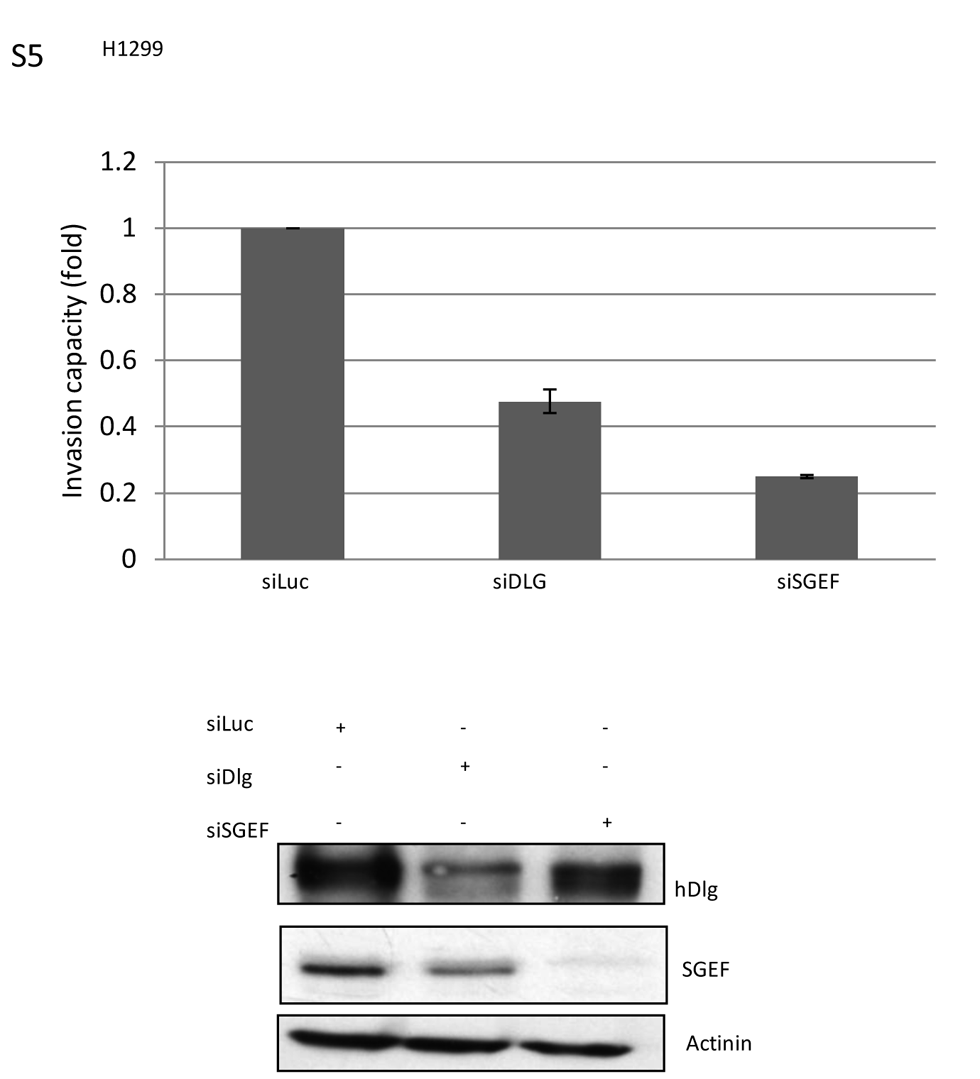

Supplement: Figure S5 — Invasive potential of H1299 cells is dependent upon hDlg and SGEF. H1299 cells were transfected with siRNAs to Luciferase (Luc), hDlg or SGEF and after 72 hrs the cells were harvested and equal numbers plated onto Matrigel invasion chambers. After overnight incubation the numbers of invading cells in the lower chamber were counted. The graph shows the fold change in the numbers of invading cells from multiple assays, where siLuc- transfected cells were scored as the reference point. Error bars represent ±SD of multiple experiments. The lower panel shows the western blot analysis of the levels of expression in total cell extracts of hDlg and SGEF following siRNA transfections performed in parallel with the invasion assays. α-Actinin is shown as the loading control. (TIF) [file ppat.1002543.s005.tif]
